# Exploring the Photodynamic Properties of Two Antiproliferative Benzodiazopyrrole Derivatives

**DOI:** 10.3390/ijms21041246

**Published:** 2020-02-13

**Authors:** Concetta Imperatore, Mohammadhassan Valadan, Luciana Tartaglione, Marco Persico, Anna Ramunno, Marialuisa Menna, Marcello Casertano, Carmela Dell’Aversano, Manjot Singh, Maria Luisa d’Aulisio Garigliota, Francesco Bajardi, Elena Morelli, Caterina Fattorusso, Carlo Altucci, Michela Varra

**Affiliations:** 1 Department of Pharmacy, University of Naples Federico II, 80131 Naples, Italy; cimperat@unina.it (C.I.); luciana.tartaglione@unina.it (L.T.); m.persico@unina.it (M.P.); mlmenna@unina.it (M.M.); marcello.casertano@unina.it (M.C.); dellaver@unina.it (C.D.); emorelli@unina.it (E.M.); 2 Department of Physics “Ettore Pancini”, University of Naples Federico II, 80126 Naples, Italy; mohammadhassan.valadan@unina.it (M.V.); manjot.singh@unina.it (M.S.); francesco.bajardi@unina.it (F.B.); 3 CoNISMa–Italian Interuniversity Consortium on Marine Sciences, Piazzale Flaminio 9, 00196 Rome, Italy; 4 Department of Pharmacy/DIFARMA, University of Salerno, 84084 Fisciano, Salerno, Italy; aramunno@unisa.it (A.R.); mdaulisiogarigliota@unisa.it (M.L.d.G.)

**Keywords:** photoswitchable azoheteroarene, diazo derivative, *cis*-*trans* conversion, fast UV spectroscopy, LC-HRMS, conformational analysis, DFT optimization

## Abstract

The identification of molecules whose biological activity can be properly modulated by light is a promising therapeutic approach aimed to improve drug selectivity and efficacy on the molecular target and to limit the side effects compared to traditional drugs. Recently, two photo-switchable diastereomeric benzodiazopyrrole derivatives **1RR** and **1RS** have been reported as microtubules targeting agents (MTAs) on human colorectal carcinoma p53 null cell line (HCT 116 p53-/-). Their IC_50_ was enhanced upon Light Emitting Diode (LED) irradiation at 435 nm and was related to their *cis* form. Here we have investigated the photo-responsive behavior of the acid derivatives of **1RR** and **1RS**, namely, *d***1RR** and *d***1RS**, in phosphate buffer solutions at different pH. The comparison of the UV spectra, acquired before and after LED irradiation, indicated that the *trans→cis* conversion of *d***1RR** and *d***1RS** is affected by the degree of ionization. The apparent rate constants were calculated from the kinetic data by means of fast UV spectroscopy and the conformers of the putative ionic species present in solution (pH range: 5.7–8.0) were modelled. Taken together, our experimental and theoretical results suggest that the photo-conversions of *trans* *d***1RR**/*d***1RS** into the corresponding *cis* forms and the thermal decay of *cis* *d***1RR**/*d***1RS** are dependent on the presence of diazonium form of *d***1RR**/*d***1RS**. Finally, a photo-reaction was detected only for *d***1RR** after prolonged LED irradiation in acidic medium, and the resulting product was characterized by means of Liquid Chromatography coupled to High resolution Mass Spectrometry (LC-HRMS) and Nuclear Magnetic Resonance (NMR) spectroscopy.

## 1. Introduction

Photo-switchable molecules have gained increasing attention in recent years, as they can provide, by means of light irradiation having a proper wavelength, external spatio-temporal control of specific events [1,2]. This important feature makes photo-switchable molecules potentially useful in a wide-range of applications ranging from molecular electronics [3,4,5] to pharmacology [6,7,8], and molecular model for visualizing interactions between biomolecules [9,10,11,12]. Azobenzenes are the most studied class of photo-switchable molecules, in view of their ability to undergo, under appropriate irradiation, a quantitative and reversible *trans-cis* isomerization [3,13]. Furthermore, depending on the intended use, azobenzenes can be easily functionalized with different chemical groups enabling a fine modulation of their photochromic responses [3,13,14,15,16]. More recently, azoheteroarenes, in which one or both the benzene rings of the azobenzene structure are replaced with one or two hetero-aromatic five-membered ring(s) have shown interesting photo-responsive properties [17,18,19].

**Figure 1:**
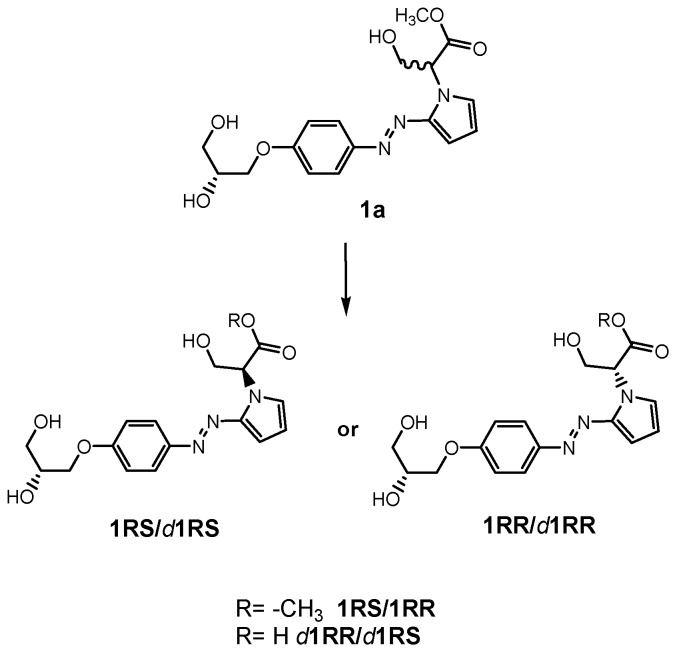
The benzodiazopyrrole derivatives previously described (the diastereomeric mixture and its pure stereomers **1RR** and **1RS**) that have shown photo-responsive antiproliferative activity against HCT 116 p53-/- cancer cells [20], and the water soluble deprotected *d***1RR** and *d***1RS** herein explored for their photo-responsive properties.

In this context, we have recently described the synthesis of some benzodiazopyrrole derivatives with photo-responsive antiproliferative activity against HCT 116 p53-/- cancer cells (Figure 1) [20]. In particular, results of the 3-(4,5-dimethylthiazol-2-yl)-2,5-diphenyltetrazolium bromide (MTT) test performed on this cancer cell line treated with the synthesized molecules and kept in the dark or under irradiation with pulsed LED at 435 nm, showed that the occurrence of the *trans-cis* isomerization is correlated with the degree of cell growth inhibition [20]. The obtained data suggested that the *cis* isomers showed the greatest antiproliferative activities. Two of these molecules forming the diastereomeric mixture named 1a (Figure 1), exhibited the most interesting biological properties. Indeed, under irradiation, the inhibition of HCT 116 p53-/- cell growth treated with 1a was greater than those treated with its pure components, **1RR** or **1RS**, thus, suggesting that the biological activity of 1a could be associated with different mechanisms of action from those induced by **1RR** and **1RS** [20]. In addition, an in vitro tubulin polymerization test also evidenced that, under LED irradiation, the two stereomers differently inhibited the tubulin aggregation into microtubules [20].

With the aim to acquire more detailed information on the physico-chemical properties that can confer to 1a its specific biological properties, and considering that the tumor aggressiveness is correlated with the lowering of the pH around the membrane of cancer cells [21,22,23,24], we have performed more in depth investigations on the photo-responsive behavior of *d***1RR** and *d***1RS** (the free carboxylic acids of **1RR** and **1RS**, Figure 1) in the pH range 8.0–5.7. To this purpose, we carried out UV and fast UV spectroscopy, and computational analyses. Furthermore, we have also explored the photo-transformation of *d***1RR** in acidic condition under prolonged LED irradiation using Liquid Chromatography High Resolution Mass Spectrometry (LC-HRMS), 1D and 2D NMR and UV techniques.

## 2. Results and Discussion

### 2.1. The Behavior of Trans/Cis Isomers of d**1RR**/d**1RS** in Differently Buffered Water-Based Solutions

**Figure 2:**
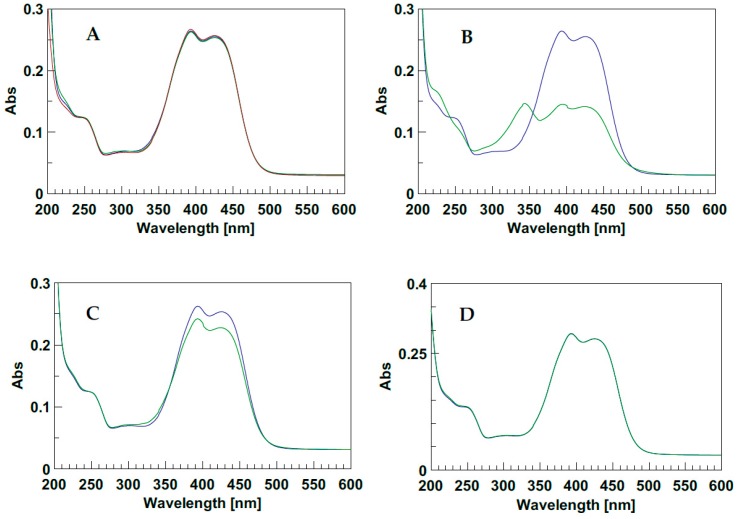
(**A**) UV spectra of *d*1RR in the dark at different pH: Red pH = 5.7; blue pH = 7.0; green pH = 8.0. (**B**–**D**) UV spectra before (blue line) and after (green line) irradiation with LED at 435 nm (1 min, 1 LED), at pH = 8.0, 7.0, and 5.7, respectively.

In order to characterize the photo-responsive properties of *d***1RR**/*d***1RS** in phosphate buffered solutions, the UV spectra of the two stereomers at different pH (8.0, 7.5, 7.0, 6.7, 6.5, 5.7) were acquired, in the dark and under LED irradiation at 435 nm. In the dark, the UV spectra of *d***1RR**/*d***1RS** showed two main, partially overlapped, UV bands centred at 394 and 424 nm (Figure 2A and Figure S1A). At pH = 8.0 and 7.0, after the irradiation of the samples, the UV profiles underwent some changes. At pH = 8.0 (Figure 2B and Figure S1B) the comparison of the two UV spectra, acquired before and after the irradiation, mostly showed a strong decreasing in the intensity of the UV bands at 394 and 424 nm and the appearance of a new band centred at 343 nm. This band, in the corresponding methyl ester derivatives **1RR**/**1RS**, has been attributed to the presence of the *cis* stereomer [20], which, according to the literature, was responsible for the differences of the light absorption before and after irradiation, due to the non-planar configuration of *cis* stereomers [13,14,15,16,17,18,19]. At pH = 7.0 only a slight decrease of the two main bands was observed in the UV spectra before and after the irradiation. A similar, but not equivalent behavior was observed for **1RR**/**1RS** in water-based solutions at pH 7.0. Indeed, in the case of *d***1RR** and *d***1RS**, the relative intensity between the band at 340 nm (*cis* isomer) and those centred around 400 nm (*trans* isomer) was significantly reduced with respect to that previously observed for **1RR**/**1RS**, possibly because an increasing of the rate of the *cis*→*trans* isomerization caused a change of the relative ratio between the concentration of the *cis*/*trans* isomers, because other species are present in solution, or both. Furthermore, this phenomenon was emphasized by increasing the pH of the buffered solution. Indeed, at pH < 7.0, there was no change in the UV band (Figure 2C,D and Figure S1C,D). Despite this, the *cis→trans* isomerization could still happen at this pH value, although it could not be monitored with normal UV spectroscopy.

**Figure 3:**
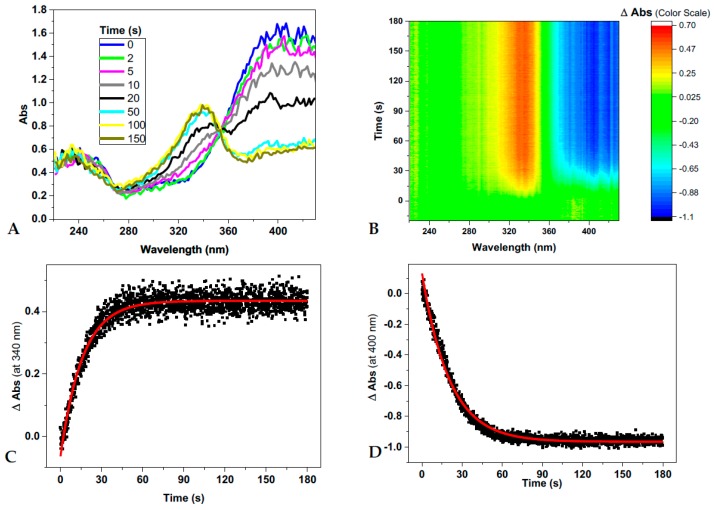
(**A**–**D**) *d***1RR** *trans* to *cis* conversion (pH = 8.0) under 5 mW laser irradiation at 435 nm. (**A**) Time-dependent absorption spectra of *d***1RR**. (**B**) 2D graphical representation of the changes of absorbance of *d***1RR** plotted as a function of time. (**C**,**D**) The changes of absorbance at specific wavelengths, 340 and 400 nm, together with their best fit (red solid lines). Reaction half-lives and apparent rate constants are deduced on these fittings.

To better explore this phenomenon, we monitored the *trans→cis* conversion of *d***1RR**/*d***1RS** under laser irradiation at 435 nm and the *cis→trans* thermal decay in the dark in buffered solutions (20 mM of phosphate buffer solutions at pH values of 5.7, 6.5, 6.7, 7.0, 7.5 and 8.0) by means of fast UV spectroscopy (a picture of this experimental set-up of the instrument is reported in Figure S2). The acquisition of time-dependent absorption spectra in the 220–430 nm range gave rise to the 2D graphical representation of time-dependent changes in absorption bands (Figure 3A,B and Figure S3A,B). Data coming from each set of measurements were processed at two wavelengths, 340 and 400 nm (Figure 3C,D and Figure S3C,D), from which the apparent rate constants for *d***1RR**/*d***1RS** *trans→cis* photo-conversions and those for the corresponding thermal relaxations were calculated (Table 1). As an example, the analysis of *d***1RR** *trans→cis* photo-conversion at pH = 8.0, and of the corresponding thermal decay in the dark, as well as those of *d***1RR**/*d***1RS** at pH 7.0 and 5.7, are shown in Figure 3 and Figures S3–S5. Under irradiation at 435 nm, a time-dependent decrease of the UV band around 400 nm (*trans* isomer) and an increase of that around 340 nm (*cis* isomer) occurred (Figure 3). As above mentioned, this observation should account for a *trans*-to-*cis* photo-isomerization. Conversely, turning off the laser irradiation, caused a time-dependent increase of the UV band at 400 nm (*trans* isomer) and a decrease of that at ≈ 340 nm (*cis* isomer) (Figure S3), due to the *cis* thermal decay. Noteworthy, the data reported in the Figures S4 and S5, showing the changes in the absorption bands centered at 340 and 400 nm for both *d***1RR**/*d***1RS** under laser irradiation and in the dark (i.e., with laser off), suggest that the *cis-trans* isomerizations always occurred at the lower pH value.

**Figure 4:**
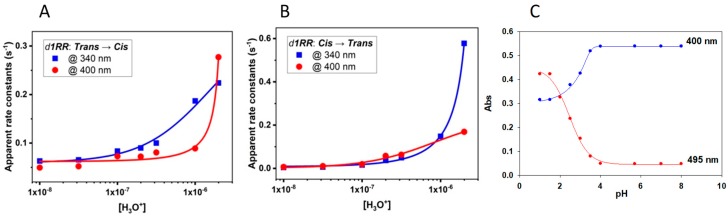
Changes in the apparent rate constants versus pH in the 5.7–8.0 range. (**A**) *d***1RR** *trans→cis* (irradiated at 435 nm). (**B**) *d***1RR** *cis→trans* (thermal decay). Both the transitions were monitored at 400 nm (circle) and 340 nm (square). Data coming from interpolations of the data are reported in Table S1. (**C**) Titration curve of *d***1RR** in phosphate buffered solution obtained plotting the absorptions at 400 and 495 nm versus pH.

All the calculated values of the apparent rate constants increase by decreasing the pH of the solutions, or also decreases by increasing the concentration of OH^−^ ions (Table 1 and Figure 3 and Figure 4). These trends showed that the *trans↔cis d***1RR**/*d***1RS** conversions exhibited the general effects of acidic catalysis, suggesting that, in water-based solutions, both the isomerizations could be affected by the acid-base ionizations [25,26,27,28,29].

At least in principle, we can consider that at very low pH both *trans*/*cis* isomers of *d***1RR**/*d***1RS** exist only in the corresponding protonated species provoking the highest possible rate for the *trans*↔*cis* conversion. In contrast, in alkaline condition, the concentration of the totally unprotonated species reaches its maximum value making lowest the rate of the *trans*↔*cis* conversions. Around the pH corresponding to the pKa value of the diazonium ions, the protonated and unprotonated species of *d***1RR**/*d***1RS** *trans*/*cis* isomers should coexist in the solution, and the apparent rate constant of the *trans*↔*cis* isomerization strongly depends on the relative abundance of the two species. Accordingly, a small variation of pH in the range containing the pKa, inducing an inversion in the relative abundance of protonated and unprotonated species, should allow a strong variation of the *trans*↔*cis* conversion rates [25,26,27,28,29]. Thus, plots of the apparent rate constants versus pH should give titration curves, from which the pKa of the diazo group in each species could be obtained. However, our data gave rise to only partial titration curves (Figure 4A,B and Figure S6A,B) since, we explored the pH/rate combinations around the physiological pH value. Despite this, the data reported in Table 1 were plotted and tentatively interpolated (Figure 4 and Figure S6) according to the method developed by Dunn et al. [27] to analyze the *trans*→*cis* isomerization of diazobenzenes in acidic medium. From the parameters of the corresponding curves (Table S1) pKa values in the range of 5.1–6.0 were obtained.

Interestingly, for both the two stereomers, *d***1RR**/*d***1RS**, the two trends of the apparent rate constants calculated from the measurements performed at 400 (a band of the *trans* isomers) and 340 nm (a band of the *cis* stereomers), as well as the trend of each absorption band measured under irradiation or during the thermal decay showed some differences (Figure 4 and Figure S6), possibly because of the differences in pKa values of the *d***1RR**/*d***1RS** *cis* and *trans* isomers (Table S1).

At lower pHs, the trend of the apparent rate constants, obtained from the measures performed at 400 and 340 nm, were also reversed comparing the two stereomers *d***1RR** (Figure 4) and *d***1RS** (Figure S6). Indeed, for *d***1RS** at pH <6.5, the apparent rate constants obtained at 400 nm increased faster than that measured at 340 nm, and the opposite trend was observed for *d***1RR**.

Accordingly, the measured absorptions at 340 and 400 nm at different pH should contain also the contribution of the UV bands of at least one specie, that is the zwitterionic form of *d***1RR**/*d***1RS**. (see the computational analyses below). The final amplitude of a specific absorption band should depend on the molar extinction coefficient and on the concentration of each species, *cis*, *trans* or the zwitterionic form, at a specific wavelength.

To further explore this issue, we performed further UV versus pH titrations on the aqueous samples of *d***1RR** and *d***1RS** buffered at pH = 8.0. At each pH variation, the UV/vis spectra of the *trans* isomers were measured (Figure S7). The obtained data showed that an additional UV band centred at about 500 nm appeared in the UV spectra of both species at pH lower than 4.5. According to literature data [25,26,27,28,29], this absorption band should be related to the corresponding diazonium ions of *d***1RR**/*d***1RS**. Each titration curve obtained plotting the absorptions measured at 400 or 495 nm versus pH supported the presence of an inflection point attributable to a pKa around 2.0-2.5 for each specie (Figure 4C and Figure S6C).

#### Computational Studies

Computational studies were performed in order to model the ionic species (ground states) of the *trans* and *cis* isomers of *d***1RR** and *d***1RS** present in solution in the pH range of 5.7–8.0.

**Scheme 1:**
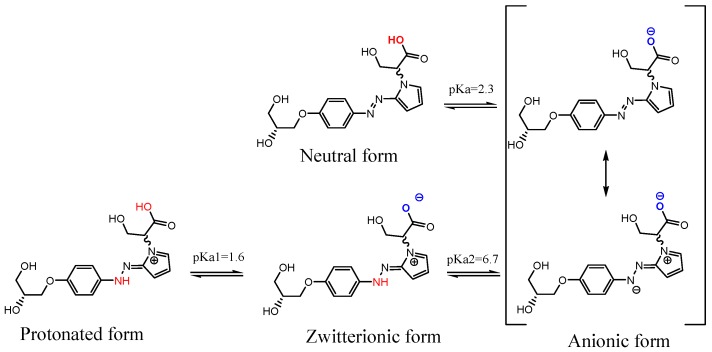
Structures of ionic forms, and pka values calculated for *d***1RR**/*d***1RS** using the ACD/Percepta software (ACD/Percepta, Advanced Chemistry Development, Inc., Toronto, ON, Canada, 2017, http://www.acdlabs.com).

At first, the apparent pKa values of *d***1RR** and *d***1RS** were calculated (ACD/Percepta, Advanced Chemistry Development, Inc., Toronto, ON, Canada, 2017, http://www.acdlabs.com). Results are reported in Scheme 1. By using the obtained pKa values, the different ratios of *d***1RR**/*d***1RS** ionic species present at the pH values considered in the UV experiments reported in Table 1, were calculated too (Table S2).

**Figure 5:**
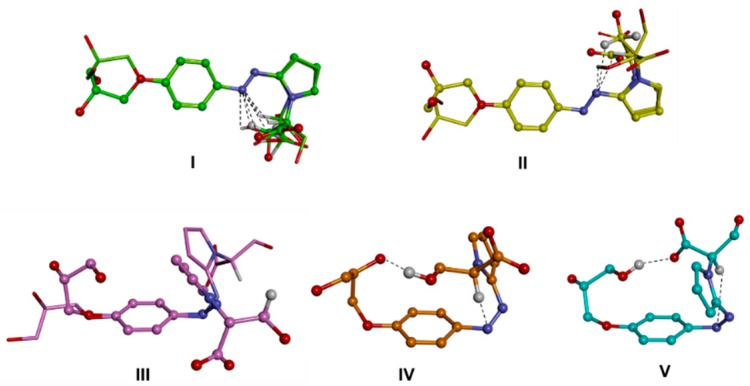
Conformational families of the *trans* (up; **I**,**II**) and *cis* (down; **III**–**V**) isomers of *d***1RR** in their anionic form. The lowest energy conformer of each family is displayed as a ball and sticks. Conformers belonging to subfamilies are superimposed by phenyl ring atoms and displayed as a stick. Heteroatoms are coloured by atom type: O, red; N, blue. Hydrogens are omitted for clarity with the exception of those involved in intramolecular interactions (evidenced with a black dashed line).

**Figure 6:**
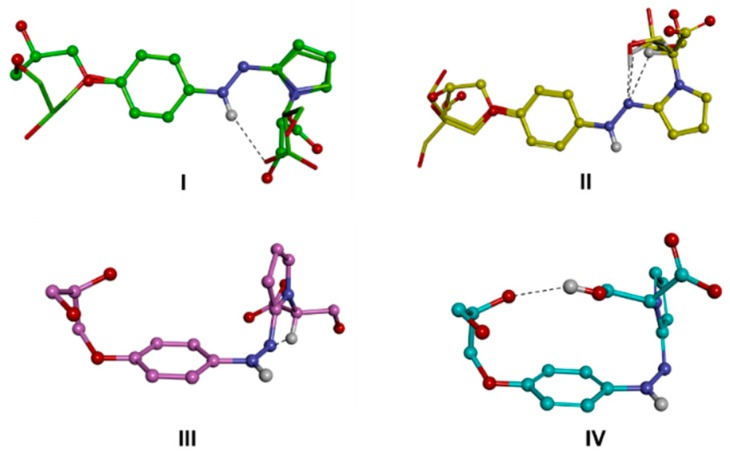
(**I**–**IV**) conformational families of the zwitterionic form of *d***1RR**. The lowest energy conformer of each family is displayed as a ball and sticks. Conformers belonging to subfamilies are superimposed by phenyl ring atoms and displayed as a stick. Heteroatoms are coloured by atom type: O, red; N, blue. Hydrogens are omitted for clarity with the exception of those involved in intramolecular interactions (evidenced with a black dashed line).

Then, the molecular models of the putative ionic species of *d***1RR** and *d***1RS** present in solution in the pH range 5.7–8.0 (i.e., the anionic forms of the *trans* and *cis* isomers and the zwitterionic form; Scheme 1; Table S2), were built and their conformational space was systematically sampled by means of molecular mechanics (MM) calculations (see the experimental section for details). The global minimum energy conformer (GM) for each species was identified, and all the other minima were ranked by their energy difference from the GM (ΔE_GM_). The conformers were grouped into conformational families according to the relative positioning of the phenyl- and pyrrole-substituted rings with respect to the diazo group (torsional angles τ1 and τ2), and further divided into sub-families according to the intramolecular interactions present within the structure (Tables S3–S8, Figure 5 and Figure 6, Figures S8 and S9). It resulted that the conformers of the *trans* isomers always presented the N=N bond and the two adjacent aromatic rings lying on the same plane (Figure 5, I–II; Figure S8, I–II; Tables S3 and S5). On the contrary, the conformers obtained for the *cis* isomers presented the phenyl- and the pyrrole-substituted rings never lying on the N=N bond plane, mostly, including the GM (sub-family IVj), presenting a *gauche* (twisted) conformation with respect to the N=N bond (Figure 5, III–V; Figure S8, III–V; Tables S4 and S6). In some minima (sub-family V_L_) the pyrrole ring is almost orthogonal with respect to the N=N bond plane. In any case, the conformers of both the *trans* and the *cis* isomers are all characterized by the presence of an intramolecular H-bond involving a nitrogen atom of the diazo group and a hydrogen atom of the substituent on the pyrrole ring.

The minima resulting from the conformational analysis on the zwitterionic form obtained after protonation (Scheme 1; Figure 6, Figure S9) assumed both the trans (families I and II) or cis (families III and IV) conformation of the NH-N bond, although with a ΔE_GM_ value about 4 kcal/mol in favour of the former. Moreover, in this case, the conformational families were characterized by different intramolecular interactions and were accordingly divided into sub-families (Tables S7 and S8). The GM of the two diastereomers resulted in the same sub-family (IId), presenting an intramolecular H-bond between the unprotonated nitrogen atom of the diazo group and the hydrogen atom on the tertiary carbon of the pyrrole substituent. Surprisingly, the conformers characterized by the ionic interaction between the two charged groups of the molecule, resulted about 4 kcal/mol from the GM in both diastereomers, likely due to the value of τ2 (Tables S7 and S8, family Im) forcing the relative positioning of the pyrrole ring with respect to the N=N bond.

Thus, to properly take into account electronic delocalization in such a conjugated system, the low energy conformers (within 5 kcal/mol from the GM) of *d***1RR** and *d***1RS** obtained by MM calculations, were, then, subjected to DFT full geometry optimization (Gaussian 09 package). The conductor-like polarizable continuum model (C-PCM) was used to mimic the presence of an aqueous solvent (see the experimental part for details). The resulting DFT structures were analyzed and ΔG_GM_ values calculated (Tables S9–S11), in addition, the length of the bonds mostly involved in electron delocalization were measured in the GM conformers (Table S12). Overall, the bond length analysis of the GM conformers indicated a higher conjugation of the diazo group with the pyrrole ring (with the bond length N-C_pyr_ closes to the double bond values) than with the phenyl-substituted ring.

The DFT GM conformers of the *trans* isomers (anionic form) resulted similar to those obtained by MM calculations (Tables S3 and S5 vs. Table S9), presenting the N=N bond and the two aromatic rings lying on the same plane, thus, allowing full electron delocalization through the π orbitals (Table S12), and an intramolecular H-bond between a nitrogen atom of the diazo group and the hydrogen atom of the secondary carbon of the substituent of the pyrrole ring. On the other contrary, the structures of the DFT conformers of the *cis* isomers resulted in significatively different from those obtained by MM calculations. As previously mentioned, overall, DFT results indicated a higher electronic conjugation with the pyrrole moiety than with the phenyl ring, contrarily to what observed for MM results (Tables S4 and S6 vs. Table S10). In particular, in the GM conformer, the substituted-pyrrole ring lays on the same plane of the N=N bond, while the substituted-benzyl ring is almost orthogonal to it, differently to what resulted from MM calculations (Tables S4 and S6 vs. Table S10). The resulting T-shaped conformation, characterized by the presence of a CH-π interaction between the pyrrole and the phenyl rings, is consistent with that obtained by previous studies [19] on azoheteroarenes where one of the aromatic moieties is an ortho mono-substituted five-membered heteroaromatic ring. Importantly, it has been observed [19] that compounds adopting a T-shaped ground state of the *cis* isomer proceed through a T-shaped isomerization pathway, increasing the stability of the *cis* isomer and its isomerization half-life.

The protonation of the anionic species was simulated by adding the proton to the anionic conformers and then performing DFT optimization of the resulting zwitterionic structure. The conformers obtained were again classified according to our families/sub-families, and the ΔG from the GM conformer was calculated (Table S11).

**Figure 7:**
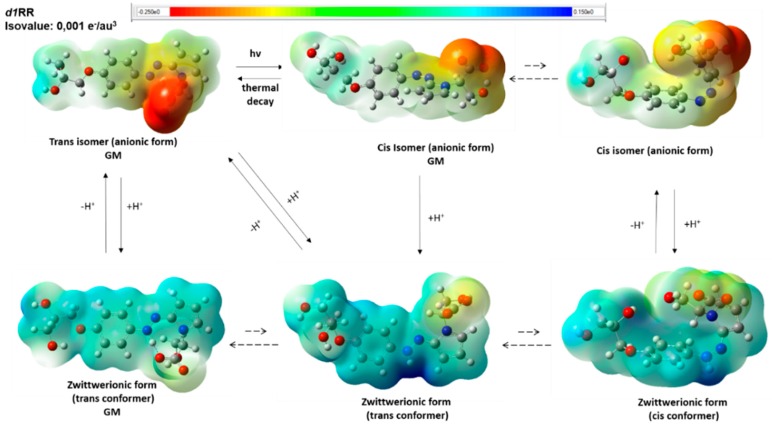
Putative *d***1RR** ionic species present in solution in the pH range considered during the fast UV experiments according to DFT calculations. Dashed arrows indicate conformational equilibria between the different conformers and GM indicate the structure of the global minimum conformer. Structures are relative to the obtained DFT minima and are displayed as a ball and sticks. Heteroatoms are coloured by atom type: O, red; N, blue. The molecular electrostatic potential surface is displayed.

Interestingly, when we added the proton to the T-shaped GM conformer of the *cis* isomer, the DFT optimized structure turned in to the zwitterionic form of the *trans* isomer (Figure 7 and Figure S10; Table S11). Thus, protonation of the *cis* anionic form in its putative prevalent T-shaped conformation led to a low energy *trans* zwitterionic conformer, which could be, then, deprotonated to the *trans* isomer (Figure 7 and Figure S10). This is in line with the disappearance of the *cis* isomer signal (345 nm) in the UV spectra when the pH is decreased from 8 to 7 (Figure 2; Table S2) and for the overall increasing of the rate of the *cis*→*trans* conversion by decreasing the pH in the fast UV experiments (Figure 4 and Figure S6).

On the other hand, the protonation of the DFT GM conformers of the *trans* anionic forms led to the GM conformer of the zwitterionic form, preserving the *trans* conformation of the parent isomer.

Taken together, our results suggest that, upon protonation (which depends on the considered pH conditions), either the *trans* and the *cis* isomers may convert into zwitterionic forms presenting a trans and planar conformation of the N-N bond with respect to the two aromatic substituents (Figure 7 and Figure S10). Importantly, contrarily to what resulted from MM calculations, the DFT GM conformer of the zwitterionic form is characterized by the presence of an ionic intramolecular interaction involving the two charged groups of the molecule (the protonated nitrogen atom and the carboxylate group; Figure 7 and Figure S10). It is reasonable to hypothesize that the activation energy necessary to break this intramolecular ionic interaction represents a factor strongly favoring the presence of the GM conformer of the (protonated) zwitterionic form.

In Figure 7 (*d***1RR**) and Figure S10 (*d***1RS**) a schematic representation of the putative equilibria involving the calculated DFT conformers of the ionic species possibly present in solutions in the 5.7–8.0 pH range, is reported.

Considering the GM structure of the zwitterionic form showed in Figure 7, it is likely that, upon carboxylate protonation, a significant amount of azonium ions would be “released” in solution. Accordingly, at pH values < 3.5, the UV data showed the appearance of an additional UV band centered at about 500 nm (Figure S7) corresponding to diazonium ions [25,26,27,28,29].

### 2.2. Photo-Induced Rearrangement of d**1RR** in a Pyridazinium Ion in Acidic Buffered Solutions: The Role of the Irradiation Exposure Time

**Figure 8:**
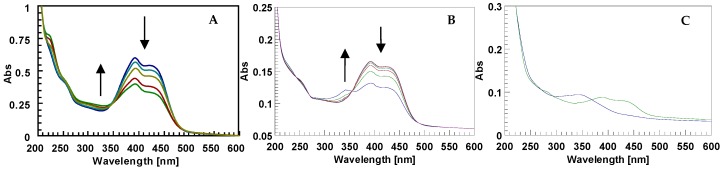
(**A**) Conversion of *d***1RR** spectrum under continuous LED irradiation at 435 nm at different exposure time (blue t = 0; light blue t = 6 h; yellow t = 12 h; red t = 15h; green t = 18 h). (**B**) *Cis-to-trans* conversion of *d***1RR** dissolved in phosphate buffered solution at pH = 8.0 after LED irradiation (12 h) at 435 nm. The arrows show the time-evolution of the UV profile in the dark, after LED irradiation. The spectra were acquired in the dark, (red line) immediately upon irradiation (blue line) and at consecutive 2-min intervals. (**C**) UV spectra of sample 1 (blue line) and of sample 2 (green line).

We observed that increasing the irradiation exposure time (LED 435 nm) of *d***1RR** in solution at pH = 5.7 led to an irreversible structural change of *d***1RR**. Indeed, after 36–48 h of pulsed (50 ms ON/500 ms OFF) or 18 h of continuous LED irradiation a strong change in the *d***1RR** UV profile appeared. In particular, the UV bands centred at 430 nm decreased in their intensity, whereas, new bands appeared in the UV 220–380 nm range (Figure 8A). Interestingly, the UV spectra acquired for *d***1RR** at different irradiation exposure times lacked the isosbestic points typical of *d***1RR** *trans→cis* conversion occurring in methanolic or alkaline conditions (Figure 8B). Furthermore, the UV spectrum of the irradiated *d***1RR** was not restored also taking the sample two months in the dark, thus, suggesting that the observed UV spectral changes were not attributable to *d***1RR** *trans→cis* conversion. In order to explore the photo-reactivity of *d***1RR** in acidic medium, the final solution was chromatographed by solid phase extraction. The column eluted with 9:1 water/methanol firstly gave two different fractions: (i) Sample 1, that showed a UV spectrum with a main band centred at about 340 nm; and (ii) sample 2 that showed a UV spectrum similar to that of *d***1RR** (Figure 8C). Both sample **1** and **2** were further analyzed by LC-HRMS.

**Figure 9:**
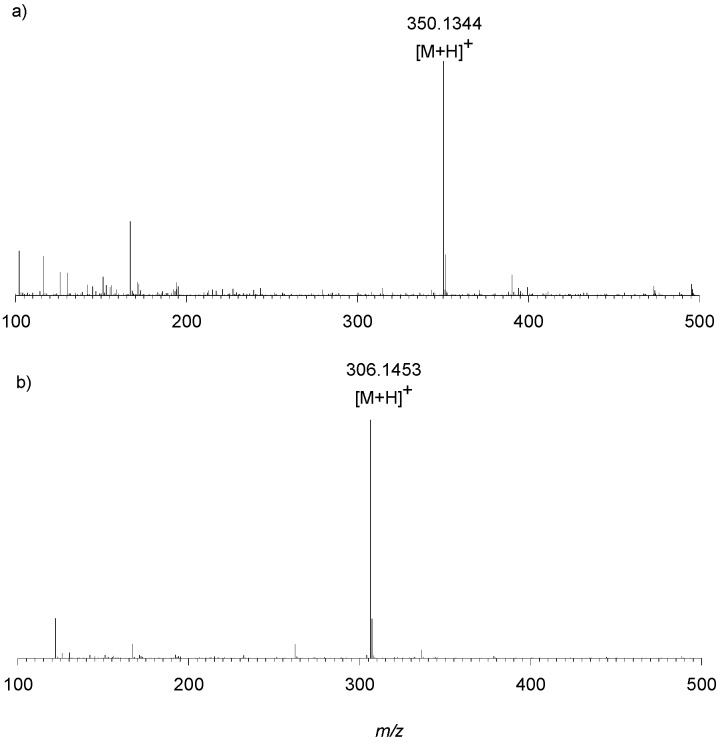
HRMS spectrum obtained by direct infusion of (**a**) *d***1RR** and (**b**) sample **1** on an LTQ Orbitrap XL™ Hybrid FT Mass Spectrometer.

Preliminary experiments on an LTQ Orbitrap XL™ Hybrid FT Mass Spectrometer system was carried out by the direct infusion of sample 1, sample 2 and *d***1RR**, with *d***1RR** used as a reference. HR full MS spectra of *d***1RR** and sample 2 appeared similar, both dominated by the [M + H]^+^ ion at *m/z* 350.1344 (C_16_H_20_N_3_O_6_^+^ Figure 9A) suggesting that sample 2 contained the unreacted *d***1RR**. On the other hand, a [M + H]^+^ ion at *m/z* 306.1453 (C_15_H_20_N_3_O_4_^+^, Figure 9B) emerged in the HR full MS spectrum of sample 1 indicating the presence of a new compound, differing from *d***1RR** for a CO_2_ portion less, that we named compound **1**. Structural features of *d***1RR** (Figure 1) reasonably lead to hypothesize that compound **1** was originated through a photo-chemical decarboxylation reaction from *d***1RR** [30].

Successively, an LC-HRMS^n^ (*n* = 1, 2) method was implemented by using a reverse-phase column and acidic mobile phases. Under the used experimental conditions, a chromatographic peak at 15.5 min. in the Extracted Ion Chromatogram (XIC) for the ion at *m/z* 350.1344 emerged for both *d***1RR** and sample 2 (Figure S11), so confirming the identity of the unmodified *d***1RR** in sample 2. Further confirmation was also obtained by the fragmentation pattern contained in the LC-HRMS^2^ spectra of *m/z* 350.1 of both *d***1RR** and sample 2 (Figures S11 and S12) that turned out to be superimposable the one to the other in containing two main diagnostic fragment ions at *m/z* 195.0770 and *m/z* 154.0503, deriving from the cleavage of the bond between diazo group and the pyrrole ring (Figure S13).

**Figure 10:**
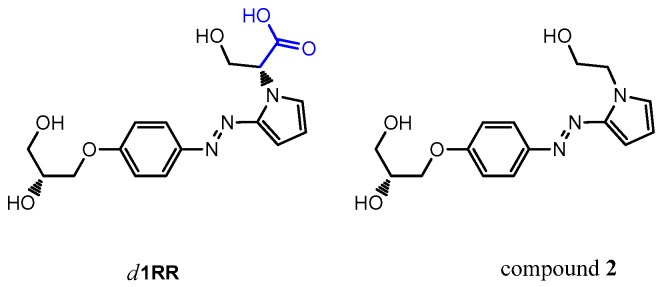
Chemical structure of *d***1RR** and of the synthesized decarboxylate derivative, named compound **2**.

A different fragmentation pattern was observed in the LC-HRMS^2^ spectrum of compound **1** (precursor ion at *m/z* 306.1, retention time = 12.7 min) dominated by a main fragment ion at *m/z* 232.1084 (C_12_H_14_N_3_O_2_^+^, Table 2) reasonably due to the cleavage of the diol linker from the phenol oxygen (neutral loss of C_3_H_6_O_2_ part structure) (Figures S14 and S15). Basing on those results, pointing out a different fragmentation behavior between compound **1** and *d***1RR**, the structural hypothesis for compound **1** being a simple decarboxylation derivative of *d***1RR**, appeared not likely. Thus, the *ex-novo* synthesis of the derivative of *d***1RR** lacking of the carboxylic group (compound **2**) was accomplished (Figure 10).

HRMS and HRMS^2^ experiments acquired for compound **2** (Figure S16) highlighted a fragmentation pattern similar to that observed for *d***1RR** (Figure S12), dominated by a main fragment ion at *m/z* 195.0773, suggesting that compound **1**, although sharing the same elemental composition with compound **2**, should be a constitutional isomer.

In order to clarify the structure of compound **1**, we performed 1D and 2D NMR spectroscopy (Figures S17–S21).

**Figure 11:**
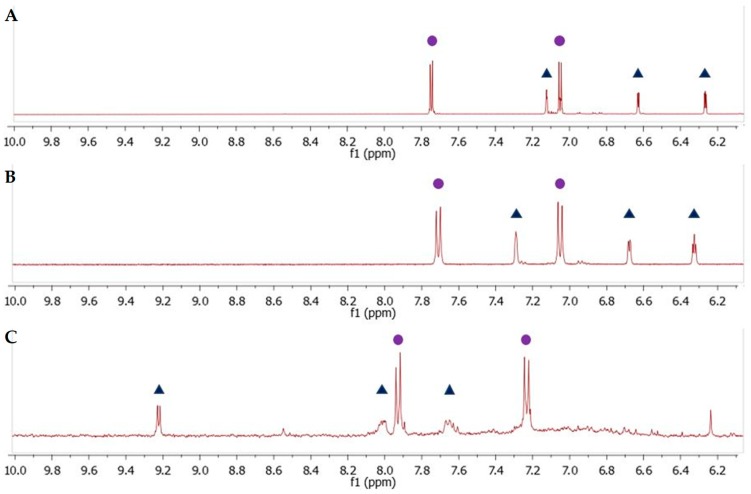
The aromatic region of ^1^H NMR spectra of compound **2** (**A**), *d***1RR** (**B**), compound **1** (**C**). Full circles show the proton on phenyl ring in all species, whereas, the triangles in (**A**,**B**) indicate the proton resonances attributable to the hydrogen on pyrrole ring and the triangles in (**C**) the pattern of new proton signals in compound **1**.

Figure 11B shows the two patterns of aromatic proton signals in *d***1RR**. The two doublets at 7.20 and 7.55 ppm are attributable to the resonances of protons on benzene ring, whereas, the three signals at 6.31, 6.65, 7.29 ppm to that on the pyrrole ring (for more detailed information see reference [20]). Only a little shift of the proton signal at 7.29 was observed in the ^1^H spectrum of compound **2** (Figure 11A and Figure S20). On the other hand, comparing ^1^H NMR data acquired on *d***1RR** with that obtained on compound **1** (Figure 11C) three main differences were observed: (i) A down-field shift of the proton resonances on benzene ring (7.98 and 7.20 ppm); (ii) the loss of the signals of the protons on pyrrole ring; (iii) a new set of three proton signals at 9.26, 8.02, and 7.62 ppm. As in the case of *d***1RR**, in the aromatic region of the ^1^H-^1^H COSY spectrum three scalar couplings (7.98 and 7.20; 9.26 and 8.02, and 8.02 and 7.62 ppm) were observed (Figure S18). These data suggested that in compound **1,** a new, deshielding, chemical environmental surrounded most of the aromatic protons.

Hence, the UV profiles, the fragmentation pattern (Table 2) and the aromatic proton resonances of compound **1** strongly suggested that the prolonging in LED irradiation time of *d***1RR** in acidic conditions promotes a rearrangement of the diazo-aromatic core of the molecule that also involves a decarboxylation reaction.

**Figure 12:**
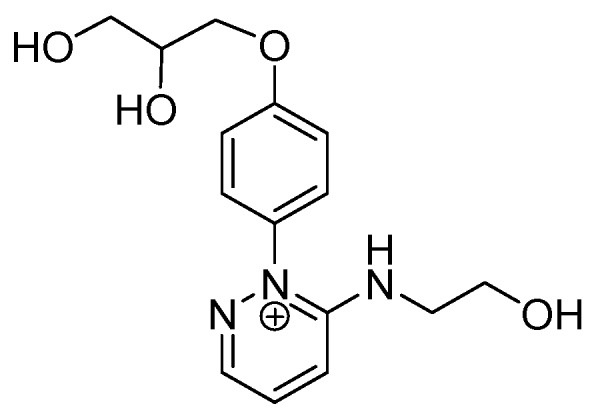
Hypothesized chemical structure of compound **1**.

We hypothesized that the diazo group and the pyrrole ring could rearrange, under irradiation, in a pyridazinium salt (Figure 12). This type of structure well fit with: (i) The blue shift of the UV band observed for compound **1 [31]** respect to *d***1RR**; (ii) the fragmentation pattern contained in HRMS^2^ spectrum (Figure S11C) and; (iii) the Δδ between the aromatic proton signals in *d***1RR** and compound **1** [32]. Despite more detailed studies should be performed to clarify better the exact reaction pathway, based on recently published data [32,33], we hypothesized that in acidic conditions a reaction similar to that described by Fehler et al. [32] could occur. Studies on the exact mechanism are currently in progress.

## 3. Materials and Methods

### 3.1. General

Commercial reagents, all organic solvents and water (HPLC grade), formic acid (95–97%, Laboratory grade), and ammonium formate (AR grade) were purchased from Sigma–Aldrich (Steinheim, Germany).

TLCs were run on Merck silica gel 60 F254 plates (Kenilworth, NJ, USA) and the spots were visualized by means of a UV lamp (Vilber Lourmat VL-4LC, 365 and 254 nm). Silica gel chromatography was performed using Merck silica gel 60 (0.063–0.200 mm). UV experiments were performed on a JASCO V-530 spectrophotometer, equipped with a PTC-348 temperature controller. ^1^H (500 MHz and 400 MHz) and ^13^C (125 MHz and 100 MHz). NMR spectra were recorded on an Agilent INOVA spectrometer (Agilent Technology, Cernusco sul Naviglio, Italy) [34,35,36]; chemical shifts were referenced to the residual solvent signal (CD_3_OD: δ_H_ = 3.31, δ_C_ = 49.0 ppm, CDCl_3_: δ_H_ = 7.26, δ_C_ = 77.0 ppm). For an accurate measurement of the coupling constants, the one-dimensional ^1^H NMR spectra were transformed at 64 K points (digital resolution: 0.09 Hz). ^1^H connectivities were determined by COSY (mixing time 100 ms).

### 3.2. Chemistry

Compounds *d***1RR** and *d***1RS** were obtained by hydrolysis of the corresponding methyl esters **1RR** and **1RS** [20] respectively (scheme S1). The synthesis of compound **2** was performed according to Scheme S1. The pyrrole derivative **VI** used in the diazo-coupling reaction was obtained from acetylation of the 2-pyrrol-1-yl-ethanol (**V**) [37]. The ^1^H and ^13^C NMR spectra of compound **2** was reported in Figures S20 and S21.

*2-Pyrrol-1-yl-ethanol* (**V**). A stirred solution of 2,5-dimethoxytetrahydrofuran (2.04 g, 15.4 mmol) in H_2_O (20 mL) was heated at reflux under N_2._ After 2h the reaction mixture was cooled to room temperature, and then a solution of ethanolamine (1.13 g, 18.5 mmol) in dichloromethane (30 mL) was added. The reaction mixture was stirred vigorously in the dark overnight, and then the organic phase was separated. The aqueous phase was re-extracted with dichloromethane (3 × 20 mL), and the combined organic layers were dried over sodium sulfate, filtered and concentrated under reduced pressure. The resulting residue was purified by flash chromatography (SiO_2_, eluted with dichloromethane) to give the title compound as a colourless oil. Yield: 62%.

^1^H NMR (400 MHz, CDCl_3_) δ: 3.86 (t, 2H, *J* = 5.2 Hz), 4.03 (t, 2H, *J* = 5.2 Hz), 6.18 (t, 2H, *J* = 2.2 Hz), 6.71 (t, 2H, *J* = 2.2 Hz). ^13^C NMR (CDCl_3_, 100 MHz) δ: 51.9, 62.9, 108.6, 120.8.

*Acetic acid 2-pyrrol-1-yl-ethyl ester* (**VI**). *N,N*-Diispropylethylamine (1.57 g, 12.1 mmol) and acetic anhydride (1.65 g, 16.2 mmol) was added to a solution of **V** (0.90 g, 8.10 mmol) in dichloromethane (10 mL), and the resulting reaction mixture was stirred at room temperature. After 15h the reaction mixture was washed with brine (2 × 20 mL), and the organic phase was dried over sodium sulfate, filtered and concentrated in vacuo. Then, the resulting residue was purified by flash chromatography (SiO_2_, dichloromethane/*n*-hexanes 4:1 as eluent) to give **VI** as a colourless oil. Yield 73%.

^1^H NMR (400 MHz, CDCl_3_) δ: 2.06 (s, 3H), 4.13 (t, 2H, *J* = 5.6 Hz), 4.32 (t, 2H, *J* = 5.6 Hz), 6.17 (t, 2H, *J* = 2.2 Hz), 6.68 (t, 2H, *J* = 2.2 Hz). ^13^C NMR (CDCl_3_, 100 MHz) δ: 20.8, 48.2, 64.0, 108.5, 120.8, 170.6.

*Compound***1**. A phosphate buffered solution of *d***1RR** at pH = 5.7 (8.0 × 10^−3^ mg/L) was irradiated for 18 h with pulse-program (50 ms-ON/1.0 min-OFF by means of an Arduino module to manage the LED plate at 435 nm). Then the solution was chromatographed on RP-18 cartridge (Oasis HLB) by means of Manifold vacuum system (Phenomenex). The cartridge was washed with water, loaded with the reaction mixture and eluted with a gradient of methanol in water. The collected fractions were analyzed by UV spectroscopy, and those containing compound **1** subjected to MS, MS^2^ spectrometry and NMR spectroscopy (Figures S14–S15, and Figures S17–S19). The same experiment was also performed using two phosphate buffered solutions of *d***1RR** at pH = 7.0 and 8.0. In these cases, no significant changes in the UV spectrum of *d***1RR** were obtained.

### 3.3. UV Spectroscopy

UV experiments were performed in phosphate buffer (0.1 M) at pH = 8.0, 7.5, 7.0, 6.7, 6.5, 6.0 and 5.7. All samples of *d***1RR** or *d***1RS** were used at a final concentration of 80 μM. o.l. cuvette 0.5 cm. In order to acquire the UV spectra before the LED irradiation, the samples were kept in the dark for 24 h. UV spectra of the *cis* isomers were acquired after irradiation of the samples at 435 nm (1 min, 1 LED 435 nm Roithner Lasertechnik). The *cis* thermal decay was monitored by the further sequential acquisition of spectra (every 1.0 min) until the UV profile became that of the initial *trans* isomer.

### 3.4. Fast UV Spectroscopy

Figure S2 reports a sketch of the experimental setup for the fast UV spectroscopy. The light source was a Deuterium arc lamp, delivering a board emission band down to about 200 nm. This acted as the probe to monitor the transient absorption spectra of the sample.

Several optical filters were used to adjust the spectrum and the average power of the probe, preventing any perturbation of the sample by the probe light. A couple of UV-grade convex lenses were used to collimate diverging output light of the lamp, and finally, it was coupled to an optical fiber that carried the light to the detector. The cuvette was kept between the two lenses, and collimated probe light passed through the sample. The detector was a spectrograph combined with an intensified CCD (ICCD), which was recording the transmitted light at a repetition rate of 10 or 25 Hz; the latter being used for the faster processes in acidic solutions.

A portion of the same samples analyzed by UV spectroscopy was used in the experiments. 1.8 mL of the sample was held in a quartz cuvette, and stirred by a magnetic stirrer. The absorption spectrum of the sample in 10 mm optical path was monitored in the 220–430 nm spectral range; resulting 2D plots of changes in absorption spectrum versus time.

Kinetic measurements were performed by means of fast UV spectroscopy for photo-conversion of the *trans* to the *cis* isomers, together with those for the reverse reactions in the dark.

### 3.5. Liquid Chromatography—High Resolution Mass Spectrometry

LC-HRMS experiments were carried out on Dionex Ultimate 3000 system which included a solvent reservoir, in-line degasser, quaternary pump and refrigerated autosampler and column oven, coupled to a hybrid linear ion trap LTQ Orbitrap XL^TM^ Fourier Transform MS (FTMS) equipped with an ESI ION MAX^TM^ source (Thermo-Fisher, San Josè, CA, USA). Chromatographic separation was accomplished by using a Poroshell 120 EC-C18, 2.7 μm, 2.1 × 100 mm column (Agilent, Santa Clara, CA, USA) maintained at room temperature and eluted at 0.2 mL/min with water (eluent A) and 95% acetonitrile/water (eluent B), both containing 2 mM ammonium formate and 50 mM formic acid. A gradient elution was used—3% B hold for the first 5 min, 3–30% B over 5 min, 30–70% B over 5 min, 70–100% B in further 5 min and hold for 4 min. Injection volume was 5 μL in all cases.

HR full MS experiments (positive ions) were carried out in the mass ranges *m/z* 100–1000 at a resolving power of 30,000 (*m/z* 400). Source settings were optimized by using *d***1RR** as reference standard and used in all the experiments: A spray voltage of 4.8 kV, a capillary temperature of 275 °C, a capillary voltage of 13 V, a sheath gas and an auxiliary gas flow of 51 and 1 (arbitrary units), respectively, and a tube lens voltage of 80 V.

HRMS^2^ data were acquired by selecting the [M + H]^+^ ion at *m/z* 350.1 and *m/z* 306.1 and fragmenting them through collision-induced dissociation (CID) with collision energy (CE) of 30% and 42%, respectively. Activation Q was set at 0.250, and an activation time of 30 msec was used.

Elemental formulae of ions contained in HRMS and HRMS^2^ spectra were assigned by using the mono-isotopic ion peak of each ion cluster and the Xcalibur 2.2 software setting a mass tolerance of 5 ppm.

### 3.6. Molecular Modeling

Molecular modeling calculations were performed on E4 Server Twin 2 × Dual Xeon-5520, equipped with two nodes. Each node: 2 × Intel^®^ Xeon^®^ QuadCore E5520-2.26Ghz, 36 GB RAM. The molecular modeling graphics were carried out on a personal computer equipped with Intel(R) Core(TM) i7-4790 processor and SGI Octane 2 workstations.

The apparent pKa values of *d***1RR** and *d***1RS** were calculated by using the ACD/Percepta software (ACD/Percepta, Advanced Chemistry Development, Inc., Toronto, ON, Canada, 2017, http://www.acdlabs.com). The percentage of neutral/ionized forms present at the pH values used in the experimental studies (i.e., range 5.7–8.0) were computed using the Handerson-Hasselbach equation. The resulting species were built and subjected to molecular mechanic (MM) energy minimization (ε = 80 × r) until the maximum RMS derivative was less than 0.001 kcal/Å, using Conjugate Gradient as minimization algorithm (Discovery Studio 2017; Dassault Systèmes BIOVIA, San Diego, 2017) [38]. Atomic potentials and charges were assigned using the CFF forcefield [39]. The conformers obtained for each compound were used as the starting structure for the subsequent systematic conformational analysis (Search Small Molecule Conformations; Discovery Studio 2017).

All the generated structures were then subjected to a systematic conformational search procedure. The conformational space was sampled by systematically varying the single bonds with an increment of 60°, as well as the double bonds with an increment of 180°. The RMSD cutoff for structure selection was set to 0.01 Å. Finally, to ensure a wide variance of the input structures to be successively fully minimized, an energy threshold value of 10^6^ kcal/mol was used as selection criteria. The generated structures were then subjected to MM energy minimization both in a vacuum and aqueous environment (ε = 1 and ε = 80 × r, respectively) until the maximum RMS derivative was less than 0.001 kcal/Å, using Conjugate Gradient as minimization algorithm. Finally, the resulting conformers were ranked by their potential energy values (i.e., ΔE from the global energy minimum (GM)). All MM conformers (ε = 80 × r) within 5 kcal/mol from the global minimum (ΔE_GM_ ≤ 5 kcal/mol) were analyzed and grouped into families, named I–V, according to the values of the dihedral angles τ1 and τ2. The resulting families were then divided into subfamilies (named a-n) on the basis of the observed intramolecular interactions.

The global minimum conformers (ε = 1 and ε = 80 × r) of each species, as well as the lowest energy conformers of the families presenting the highest number of conformers (ε = 80 × r) have been subjected to DFT full geometry optimization. The calculations were carried out using the Gaussian 09 package [40]. All structures were fully optimized at the B3LYP/6-31+G(d,p) level [41,42] using the conductor-like polarizable continuum (C-PCM) model, which allows the calculation of the energy in the presence of a solvent [43]. In this case, all structures were optimized as a solute in an aqueous solution. In order to characterize every structure as minimum and to calculate the Gibbs free energy, a vibrational analysis was carried out at the same level of theory using the keyword freq. The RMS force criterion was set to 3 × 10^−4^ a.u. Partial charges have been calculated using the natural bond orbital (NBO) method [44].

## 4. Conclusions

Through the use of UV and UV fast spectroscopy the kinetic behavior of the *trans*↔*cis* isomerizations of two azoheteroarenes, named *d***1RR**/*d***1RS** has been studied, using phosphate (0.1M) solutions buffered at different pH (from 8.0 to 5.7). The results evidenced at least two important features.

First of all, the increasing of the proton concentration provoked a general increase in the rates of both the photo-conversions of *trans d***1RR**/*d***1RS** into the corresponding *cis* forms and of the thermal decay of *cis d***1RR**/*d***1RS**. According to literature data, the protonation of the diazo group of *d***1RR**/*d***1RS** should occur in the solution [25,26,27,28,29].

The computational analysis of the conformational preference of the calculated species present in solution in the pH range 5.7–8.0 highlighted that while the *trans* isomer preferentially adopts a fully planar and conjugated conformation, the *cis* isomer is characterized by a T-shaped conformation. Importantly, it has been reported that azoheteroarene photoswitches able to adopt a T-shaped ground state conformation of the *cis* isomer are characterized by longer half-lives [19]. Noteworthy, upon protonation both the *trans* and the *cis* isomers resulted in the formation of a zwitterionic form characterized by a trans and planar conformation and stabilized by ionic intramolecular interaction between the two charged groups. Thus, our results indicate that protonation of *d***1RR**/*d***1RS** can strongly affect their ground state conformation increasing the rate of *trans*↔*cis* isomerizations. Second, the results of fast UV experiments also showed that for both of the two stereomers, *d***1RR**/*d***1RS**, the pKa values of the *cis* and *trans* isomers should be slightly different. Indeed, the kinetic measurements performed at 400 (a band of the *trans* isomers) and 340 nm (a band of the *cis* stereomers) gave rise to two different trends of the apparent rate constants versus pH.

The investigation on *d***1RR**/*d***1RS** photo-responsive properties also showed that *d***1RR**, but not *d***1RS** underwent to a photo-transformation under prolonged irradiation. The characterization of the final molecule produced in this condition led to the identification of a new pyridazinium salt. Interestingly, the pyridazinium salt is formed only from highly diluted solution buffered at pH = 5.7, thus, suggesting that the mechanism of the reaction should be monomolecular and that the reaction should be promoted by the presence of *d***1RR** diazonium ions in solution. Despite further studies are necessary to establish the exact way of this reaction, the different behavior of *d***1RR** and *d***1RS** under prolonged reaction herein reported could be the reason of the previously reported different dose-response antiproliferative action of **1RR**, **1RS** and their mixture [20].

## Figures and Tables

**Table 1:** Apparent rate constants calculated in s^−1^ from kinetic data by means of fast UV spectroscopy. The reported values have about 10% uncertainty.

	*1RR*				*1RS*			
pH	*trans* → *cis* *hν*		*cis* → *trans* Δ		*trans* → *cis* *hν*		*cis* → *trans* Δ	
	340 nm	400 nm	340 nm	400 nm	340 nm	400 nm	340 nm	400 nm
8.0	6.30 × 10^−^^2^	4.95 × 10^−2^	4.18 × 10^−3^	6.19 × 10^−3^	6.86 × 10^−2^	5.64 × 10^−2^	3.69 × 10^−3^	5.10 × 10^−3^
7.5	6.54 × 10^−2^	5.21 × 10^−2^	6.08 × 10^−3^	1.03 × 10^−2^	7.38 × 10^−2^	5.97 × 10^−2^	6.66 × 10^−3^	9.22 × 10^−3^
7.0	8.35 × 10^−2^	7.30 × 10^−2^	1.57 × 10^−2^	1.93 × 10^−2^	7.79 × 10^−2^	6.48 × 10^−2^	1.48 × 10^−2^	2.10 × 10^−2^
6.7	9.00 × 10^−2^	7.22 × 10^−2^	3.65 × 10^−2^	5.77 × 10^−2^	9.50 × 10^−2^	7.70 × 10^−2^	3.85 × 10^−2^	5.33 × 10^−2^
6.5	0.100	8.06 × 10^−2^	4.95 × 10^−2^	6.30 × 10^−2^	9.00 × 10^−2^	7.96 × 10^−2^	4.95 × 10^−2^	6.03 × 10^−2^
6.0	0.187	8.89 × 10^−2^	0.148	0.239	0.116	0.210	0.131	0.148
5.7	0.224	0.277	0.578	0.169	0.224	0.385	0.210	0.347

**Table 2:** Assignment of fragment ions contained in the HR CID MS^2^ spectrum of compound **1**. Elemental formulae of the mono-isotopic ion peaks (*m/z*) are reported with double bond/ring equivalents (RDB) and errors (Δ, ppm).

Exp. MS (*m/z*)	Δ ppm, RDB	Formula	Structural hypothesis
**Precursor ion**			
306.1457	2.833, 7.5	C_15_H_20_N_3_O_4_^+^	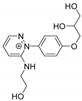
**Fragment ions**			
288.1351	2.888, 8.5	C_15_H_18_N_3_O_3_^+^	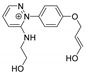
275.1274	3.479, 8.0	C_14_H_17_N_3_O_3_^•+^	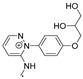
232.1084	1.494, 7.5	C_12_H_14_N_3_O_2_^+^	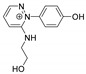
262.1193	2.602, 7.5	C_13_H_16_N_3_O_3_^+^	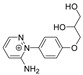
247.1083	2.352, 7.05	C_13_H_15_N_2_O_3_^+^	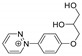
220.0975	3.090, 6.5	C_12_H_14_NO_3_^+^	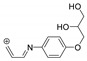
214.0981	2.856, 8.5	C_12_H_12_N_3_O^+^	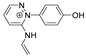
204.1138	3.240, 6.5	C_11_H_14_N_3_O^+^	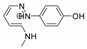
188.0824	2.985, 7.5	C_10_H_10_N_3_O^+^	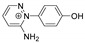
173.0714	2.661, 7.5	C_10_H_9_N_2_O^+^	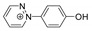
159.0683	2.732, 7.0	C_10_H_9_NO^•+^	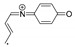
146.0604	2.462, 6.5	C_9_H_8_NO^+^	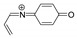
108.0445	1.015, 4.5	C_6_H_6_NO^+^	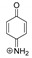

## Supplementary Materials

Supplementary materials can be found at https://www.mdpi.com/1422-0067/21/4/1246/s1. Figure S1. UV spectra of *d***1RS** in the dark at different pH, Figure S2. A schematic representation of the instrument employed for fast UV spectroscopy, Figure S3. *Cis* to *trans* thermal conversion for *d***1RR**, Figure S4. *Cis* to *trans* and *trans* to *cis* conversion for *d***1RR** at pH 5.7 and 7.0, Figure S5. *Cis* to *trans* and *trans* to *cis* conversion for *d***1RS** at pH 5.7 and 7.0, Figure S6. Changes in apparent rate constant (*k*_obs_) in the pH range 5.7–8.0, Figure S7. UV-vis spectra of *d***1RR** and *d***1RS** in the pH range 7.0–1.5, Figure S8. Conformational families of *trans* (I-II) and *cis* (III-V) isomers of *d***1RS**, Figure S9. Conformational families of the zwitterionic form of *d***1RS**, Figure S10. Putative *d***1RS** ionic species present in solution, Figure S11. LC-HRMS of sample **2**: (**A**) Extracted ion Chromatogram at *m/z* 350.1344, (**B**) associated HRMS spectrum and (**C**) associated HRMS^2^ spectra, Figure S12. HRMS^2^ spectrum of *d***1RR** obtained by using the ion at *m/z* 350.1 as precursor, Figure S13. The cleavage occurring for *d***1RR** in HRMS^2^ experiments giving rise to two main fragment ions, Figure S14. LC-HRMS of compound **1**: (**A**) Extracted ion Chromatogram at *m/z* 306.1453 (**B**) associated HRMS and (**C**) HRMS^2^ spectra, Figure S15. Enlargement range of the LC-HRMS^2^ spectrum of the precursor ion of compound **1**, Figure S16. HRMS and HRMS^2^ spectra of compound **2**, Figure S17. ^1^H NMR spectrum of compound **1** in CD_3_OD, Figure S18. Low-field enlargement of ^1^H-^1^H COSY NMR spectrum of compound **1** in CD_3_OD, Figure S19. High-field enlargement of ^1^H-^1^H COSY NMR spectrum of compound **1** in CD_3_OD, Figure S20. ^1^H NMR spectrum of compound **2** in CD_3_OD. Figure S21, ^13^C NMR spectrum of compound **2** in CD_3_OD, Table S1. Data coming from the interpolation of the apparent rate constants versus [H^+^], Table S2. Calculated percentages of ionic species of *d***1RR** and *d***1RS** in the pH range 1.0-8.0, Table S3. ΔE_GM_ values and torsion angle values of the MM conformers of *trans* isomer of *d***1RR**, Table S4. ΔE_GM_ values and torsion angle values of the MM conformers of *cis* isomer *d***1RR**, Table S5. ΔE_GM_ values and torsion angle values of the MM conformers of *trans* isomer of *d***1RS**, Table S6. ΔE_GM_ values and torsion angle values of the MM conformers of *cis* isomer *d***1RS**, Table S7. ΔE_GM_ values and torsion angle values of the MM conformers of *d***1RR** in the zwitterionic form, Table S8. ΔE_GM_ values and torsion angle values of the MM conformers of *d***1RS** in the zwitterionic form, Table S9. ΔG_GM_ values and torsion angle values of the DFT *trans* isomers of ***d*1RS** and *d***1RR** in the anionic form, Table S10. ΔG_GM_ values and torsion angle values of the DFT *cis* isomer of *d***1RS** and *d***1RR** in the anionic form, Table S11. ΔG_GM_ values and torsion angle values of the DFT conformers of *d***1RS** and *d***1RR** in the zwitterionic form, Table S12. Bond length values (Å) of the DFT conformers of *d***1RR** and *d***1RS**, Scheme S1. Synthesis of compound **2**.
