# Supplementary material for: Exploring the Photodynamic Properties of Two Antiproliferative Benzodiazopyrrole Derivatives
Source: Int J Mol Sci. 2020 Feb 13;21(4):1246. doi: 10.3390/ijms21041246 (PMC7072997; doi:10.3390/ijms21041246)
Supplement: Supplementary file 1 [file ijms-21-01246-s001.pdf]

Supplementary materials for

Exploring the photodynamic properties of two antiproliferative benzodiazopyrrole derivatives

Concetta Imperatore†1, Mohammadhassan Valadan†2, Luciana Tartaglione[1,3], Marco Persico[1], Anna Ramunno[4], Marialuisa Menna[1], Marcello Casertano[1], Carmela Dell'Aversano[1,3], Manjot Singh[2], Maria Luisa d'Aulisio Garigliota[4], Francesco Bajardi[2], Elena Morelli[1], Caterina Fattorusso*1, Carlo Altucci*2 and Michela Varra*1

[1] Department of Pharmacy, University of Naples Federico II, Naples 80131, Italy.; cimperat@unina.it (C.I.); luciana.tartaglione@unina.it (L.T.); m.persico@unina.it (M.P.); mlmenna@unina.it (M.M.); marcello.casertano@unina.it (M.C.); dellaver@unina.it (C.D.A.); emorelli@unina.it (E.M.);

[2] Department of Physics "Ettore Pancini", University of Naples Federico II, Naples 80126, Italy; mohammadhassan.valadan@unina.it (M.V.); singh@fisica.unina.it (M.S.); fbajardi@fisica.unina.it (F.B.);

[3] CoNISMa – Italian Interuniversity Consortium on Marine Sciences, Piazzale Flaminio 9, 00196 Rome, Italy

[4] Department of Pharmacy/DIFARMA, University of Salerno, 84084 Fisciano, Salerno, Italy; aramunno@unisa.it (A.R.); mdaulisiogarigliota@unisa.it (M.L.D.A.G.)

* Correspondence: varra@unina.it (Mi.V.), Tel.: +39-081-678540 (M.V.); altucci@na.infn.it (C.A.), Tel.: +39-081-676293; caterina.fattorusso@unina.it (C.F.) Tel.: +39-081-678544

† These authors contributed equally to this work.

TABLE OF CONTENTS

Figure S1. UV spectra of *d***1RS** in the dark at different pH 3

Figure S2. A schematic representation of the instrument employed for fast UV spectroscopy 4

Figure S3. *Cis* to *trans* thermal conversion for *d***1RR** 5

Figure S4. *Cis* to *trans* and *trans* to *cis* conversion for *d***1RR** at pH 5.7 and 7.0 6

Figure S5. *Cis* to *trans* and *trans* to *cis* conversion for *d***1RS** at pH 5.7 and 7.0 7

Figure S6. Changes in apparent rate constant (k_{obs}) in the pH range 5.7-8.0 8

Figure S7. UV-vis spectra of *d***1RR** and *d***1RS** in the pH range 7.0-1.5 8

Figure S8. Conformational families of *trans* (I-II) and *cis* (III-V) isomers of *d***1RS** 9

Figure S9. Conformational families of the zwitterionic form of *d***1RS** 9

Figure S10. Putative *d***1RS** ionic species present in solution 10

Figure S11. LC-HRMS of sample **2**: (**A**) Extracted ion Chromatogram at *m*/*z* 350.1344, (**B**) associated HRMS spectrum and (**C**) associated $HRMS^2$ spectra 11

Figure S12. $HRMS^2$ spectrum of *d***1RR** obtained by using the ion at *m*/*z* 350.1 as precursor 13

Figure S13. The cleavage occurring for *d***1RR** in $HRMS^2$ experiments giving rise to two main fragment ions 13

Figure S14. LC-HRMS of compound **1:** (**A**) Extracted ion Chromatogram at *m*/*z* 306.1453 (**B**) associated HRMS and (**C**) $HRMS^2$ spectra 14

Figure S15. Enlargement range of the LC-$HRMS^2$ spectrum of the precursor ion of compound **1** 16

Figure S16. HRMS and $HRMS^2$ spectra of compound **2** 17

Figure S17. ^{1}H NMR spectrum of compound **1** in CD_3OD 18

Figure S18. Low-field enlargement of ^{1}H-^{1}H COSY NMR spectrum of compound **1** in CD_3OD 19

Figure S19. High-field enlargement of ^{1}H-^{1}H COSY NMR spectrum of compound **1** in CD_3OD 20

Figure S20. ^{1}H NMR spectrum of compound **2** in CD_3OD 21

Figure S21. ^{13}C NMR spectrum of compound **2** in CD_3OD 22

Table S1. Data coming from the interpolation of the apparent rate constants versus [H^+] 23

Table S2. Calculated percentages of ionic species of *d***1RR** and *d***1RS** in the pH range 1.0-8.0 24

Table S3. ΔE_{GM} values and torsion angle values of the MM conformers of *trans* isomer of *d***1RR** 25

Table S4. ΔE_{GM} values and torsion angle values of the MM conformers of *cis* isomer *d***1RR** 26

Table S5. ΔE_{GM} values and torsion angle values of the MM conformers of *trans* isomer of *d***1RS** 27

Table S6. ΔE_{GM} values and torsion angle values of the MM conformers of *cis* isomer *d***1RS** 28

Table S7. ΔE_{GM} values and torsion angle values of the MM conformers of *d***1RR** in the zwitterionic form 29

Table S8. ΔE_{GM} values and torsion angle values of the MM conformers of *d***1RS** in the zwitterionic form 30

Table S9. ΔG_{GM} values and torsion angle values of the DFT *trans* isomers of ***d*1RS** and *d***1RR** in the anionic form 31

Table S10. ΔG_{GM} values and torsion angle values of the DFT *cis* isomer of *d***1RS** and *d***1RR** in the anionic form 32

Table S11. ΔG_{GM} values and torsion angle values of the DFT conformers of *d***1RS** and *d***1RR** in the zwitterionic form 33

Table S12. Bond length values (Å) of the DFT conformers of *d***1RR** and *d***1RS** 34

Scheme S1. Synthesis of compound **2** 35

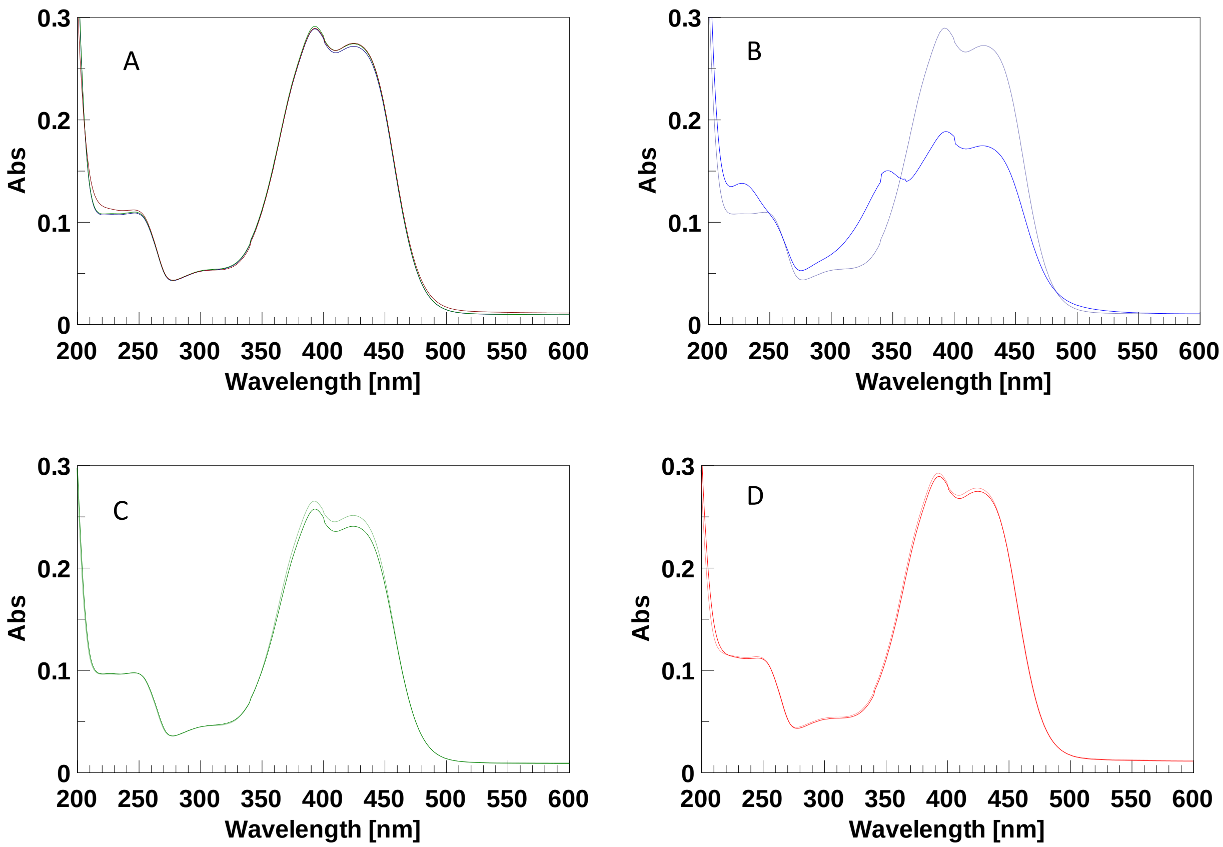

Figure S1. **A**. UV spectra of *d***1RS** in the dark at different pH: red pH=5.7; blue pH=7.5; green pH= 8.0. **B**, **C**, and **D**. UV spectra before (dashed line) and after (blue line) irradiation with LED at 435 nm (1 min, 1 LED), at pH=8.0, 7.5, and 5.7 respectively.

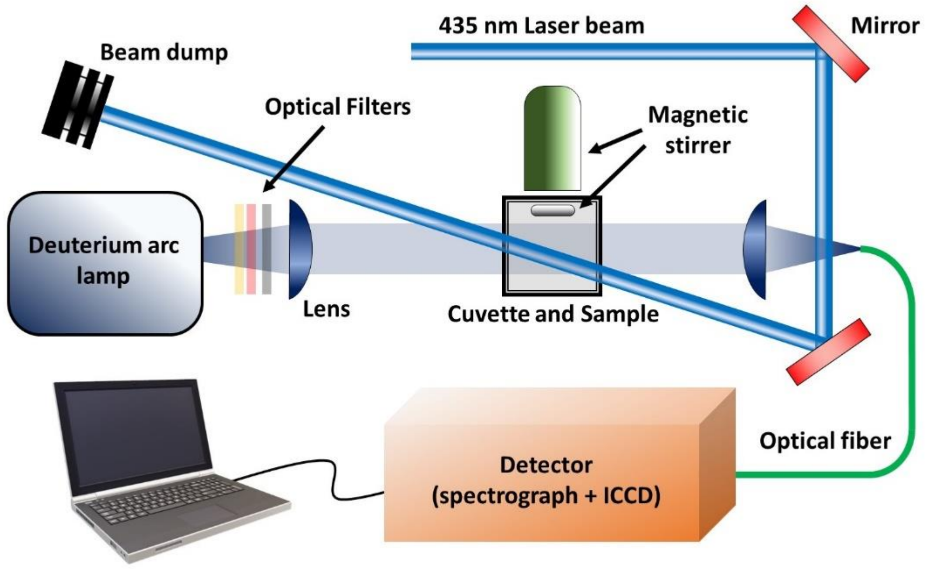

Figure S2. The experimental setup for the fast UV spectroscopy. The light source is a Deuterium arc lamp, delivering a board emission band down to about 200 nm. This acts as the probe to monitor transient absorption spectra of the sample. Several optical filters are used to adjust the spectrum and the average power of the probe, preventing any perturbation of the sample by the probe light. A couple of UV-grade convex lenses are used to collimate diverging output light of the lamp and finally couple it to an optical fiber that carries the light to the detector. The cuvette is kept between the two lenses and collimated probe light passes through the sample. The detector is a spectrograph combined with an intensified CCD (ICCD), which is recording the transmitted light at a repetition rate of 10 or 25 Hz; the latter being used for the faster processes in acidic solutions.

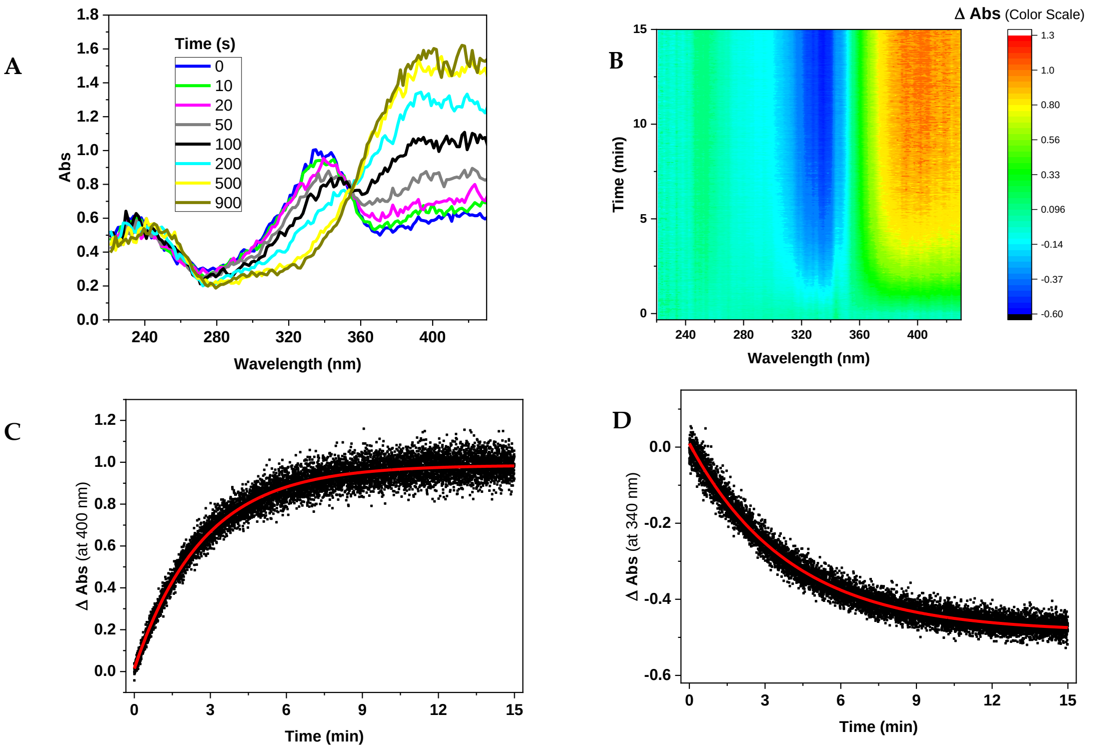

Figure S3. Fast UV spectroscopy of *d***1RR** at pH=8.0. **A** and **B**. *Cis* to *trans* thermal conversion (in the dark pH=8.0) for *d***1RR** and **A.** Time-dependent absorption spectrum of *d***1RR**. **B**. 2D graphical representation of the changes in the absorbance of *d***1RR** plotted as a function of time. **C** and **D.** The changes of absorbance at specific wavelengths 340 and 400 nm, together with their best fit (red solid lines). Reaction half-lives and apparent rate constants are deduced based on these fittings.

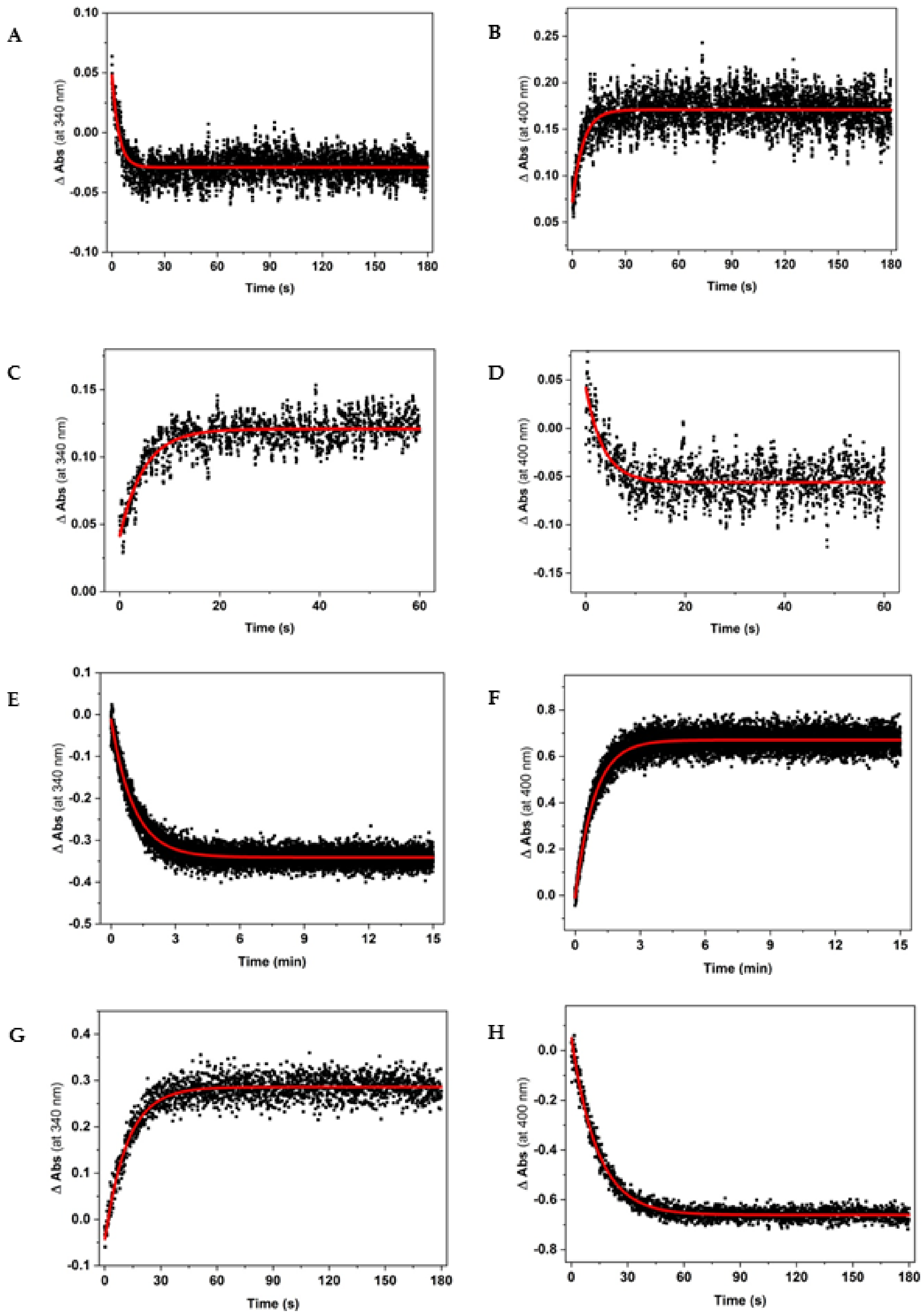

Figure S4. Some examples of the changes of absorbance at specific wavelengths 340 and 400 nm, measured by fast UV spectroscopy, together with their best fit (red solid lines). *d***1RR** *cis→trans* (**A** and **B**) and *trans→cis* (**C** and **D**) conversion at pH=5.7. *d***1RR** *cis→trans* (**E** and **F**) and *trans→cis* (**G** and **H**) conversion at pH= 7.0. Reaction half-lives and apparent rate constants are deduced based on these fittings.

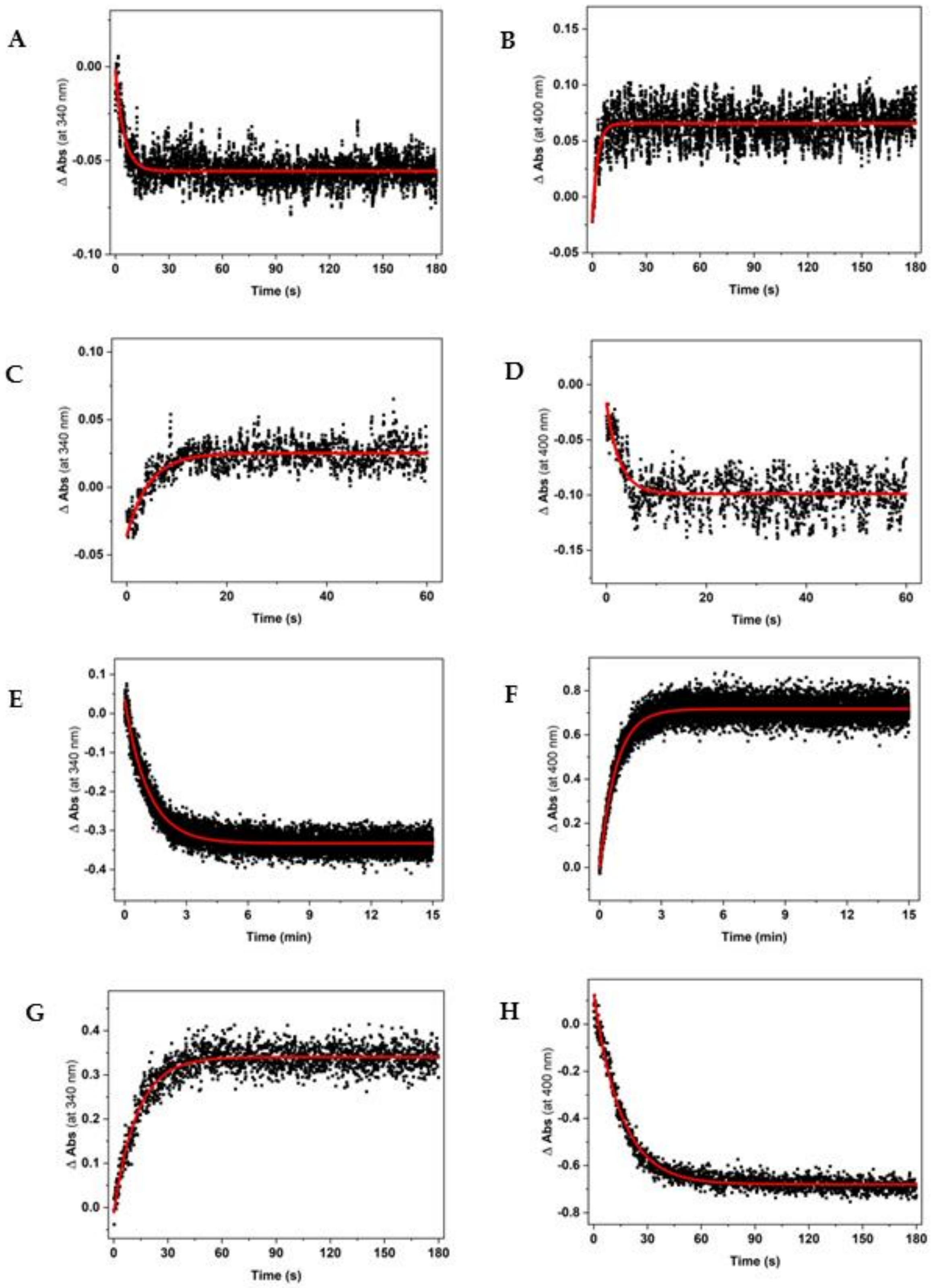

Figure S5. Some examples of the changes of absorbance at specific wavelengths 340 and 400 nm, measured by fast UV spectroscopy, together with their best fit (red solid lines). *d***1RS** *cis→trans* (**A** and **B**) and *trans→cis* (**C** and **D**) conversion at pH=5.7. *d***1RS** *cis→trans* (**E** and **F**) and *trans→cis* (**G** and **H**) conversion at pH= 7.0. Reaction half-lives and apparent rate constants are deduced based on these fittings.

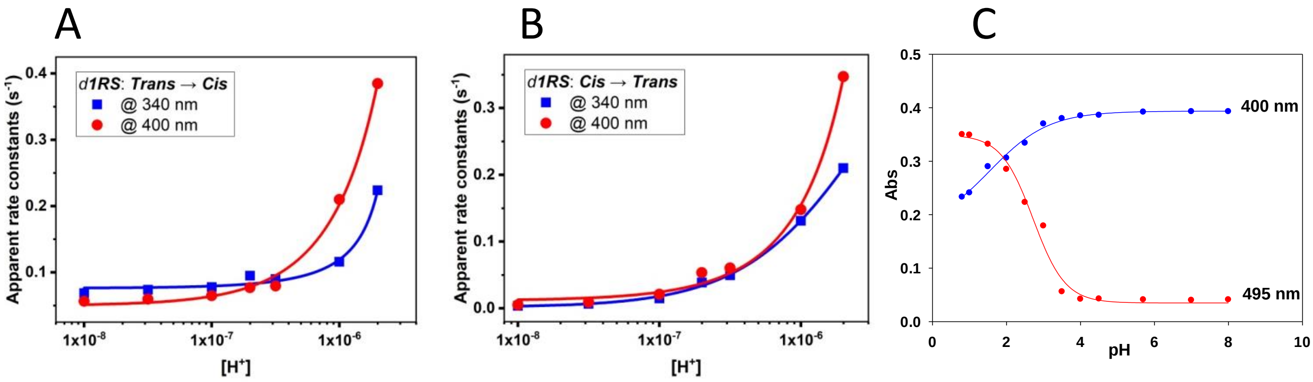

Figure S6. Changes in apparent rate constant (k_{obs}) in the pH range 5.7-8.0. **A**. *d***1RS** *trans→cis* (irradiated at 435 nm). **B**. *d***1RS** *cis→trans* (thermal decay). Both the transitions were monitored at 400 nm (circle) and 340 nm (square). **C**. Titration curve of *d***1RS** in phosphate buffered solution obtained plotting the absorptions at 400 and 495 nm *versus* pH.

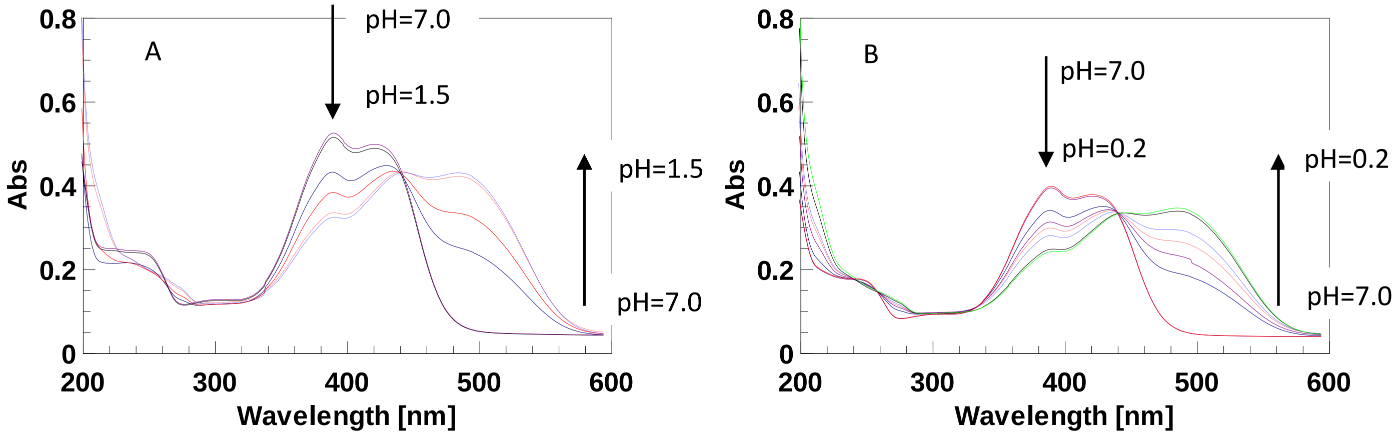

Figure S7. UV spectra of *d***1RR** (**A**) and *d***1RS** (**B**) acquired at different pH values. The arrows show the behaviours of the UV bands at different pH values (Panel A: 7.0, 5.5, 4.5, 3.5, 2.5, 1.5; Panel B: 7.0, 5.5, 4.5, 3.5, 2.5, 1.5, 0.5, 0.2)

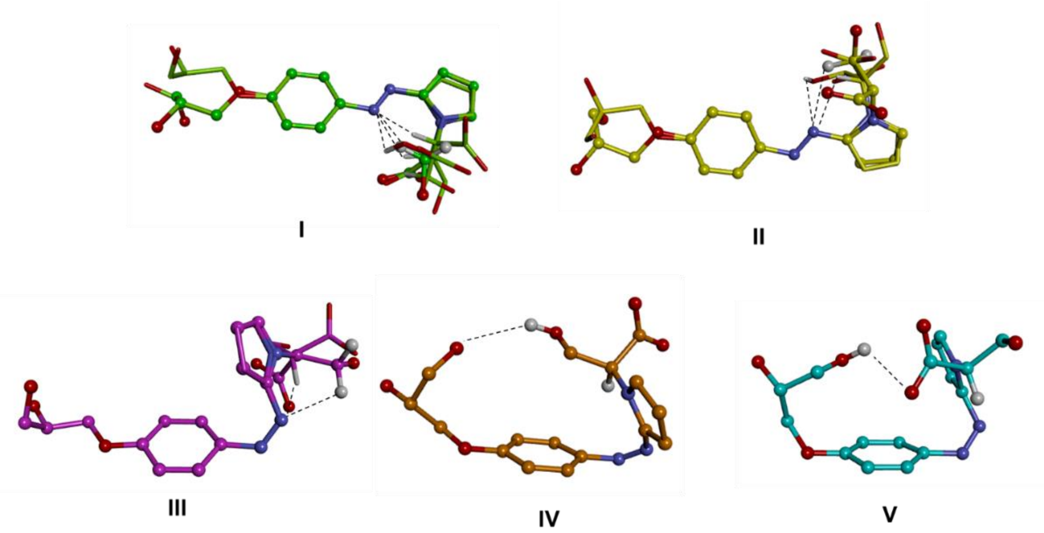

Figure S8. Conformational families of the *trans* (up; I and II) and *cis* (down; III-V) isomers of *d***1RS** in their anionic form. The lowest energy conformer of each family is displayed as ball & sticks. Conformers belonging to subfamilies are superimposed by phenyl ring atoms and displayed as stick. Heteroatoms are coloured by atom type: O, red; N, blue. Hydrogens are omitted for clarity with the exception of those involved in intramolecular interactions (evidenced with a black dashed line).

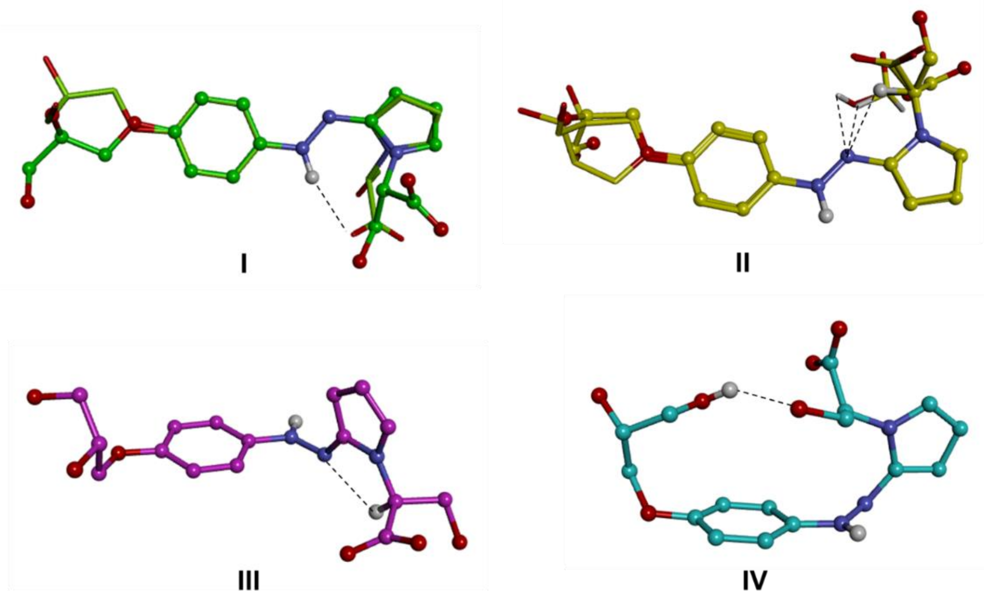

Figure S9. Conformational families of the zwitterionic form of *d***1RS.** The lowest energy conformer of each family is displayed as ball & sticks. Conformers belonging to subfamilies are superimposed by phenyl ring atoms and displayed as stick. Heteroatoms are coloured by atom type: O, red; N, blue. Hydrogens are omitted for clarity with the exception of those involved in intramolecular interactions (evidenced with a black dashed line).

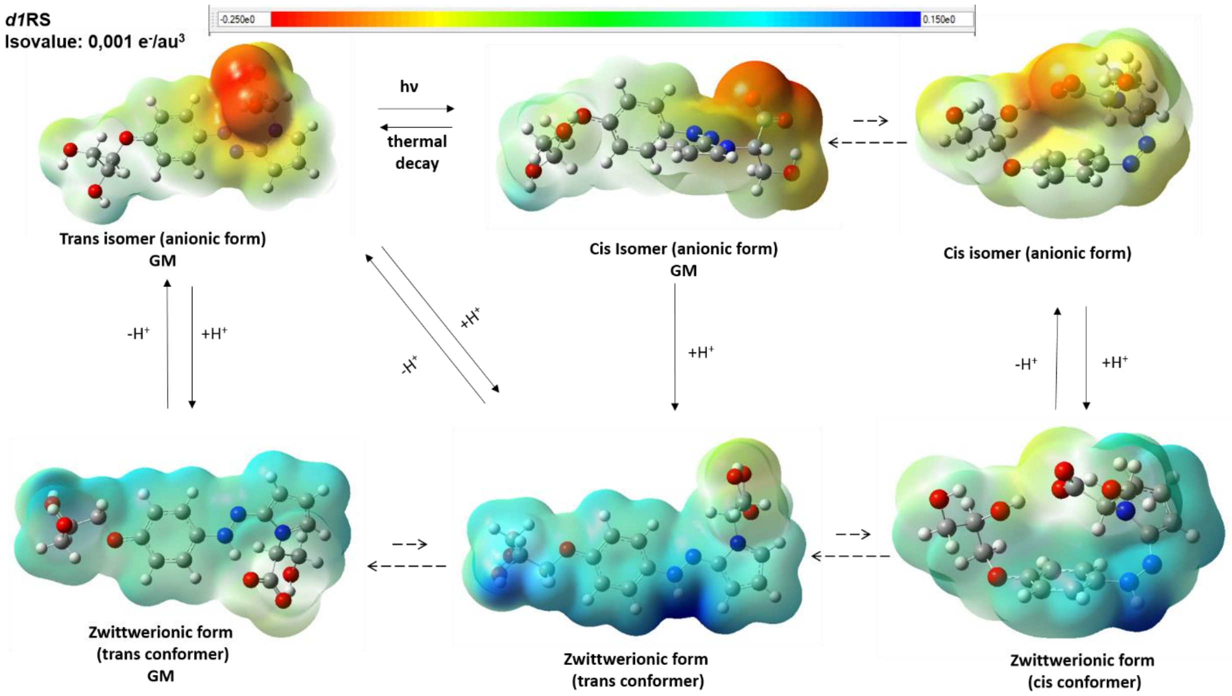

Figure S10. Putative *d*1**RS** ionic species present in solution in the pH range considered during the fast UV experiments according to DFT calculations. Dashed arrows indicate conformational equilibria between the different conformers and GM indicate the structure of the global minimum conformer Structures are relative to the obtained DFT minima and are displayed as ball & sticks. Heteroatoms are coloured by atom type: O, red; N, blue. The molecular electrostatic potential surface is displayed.

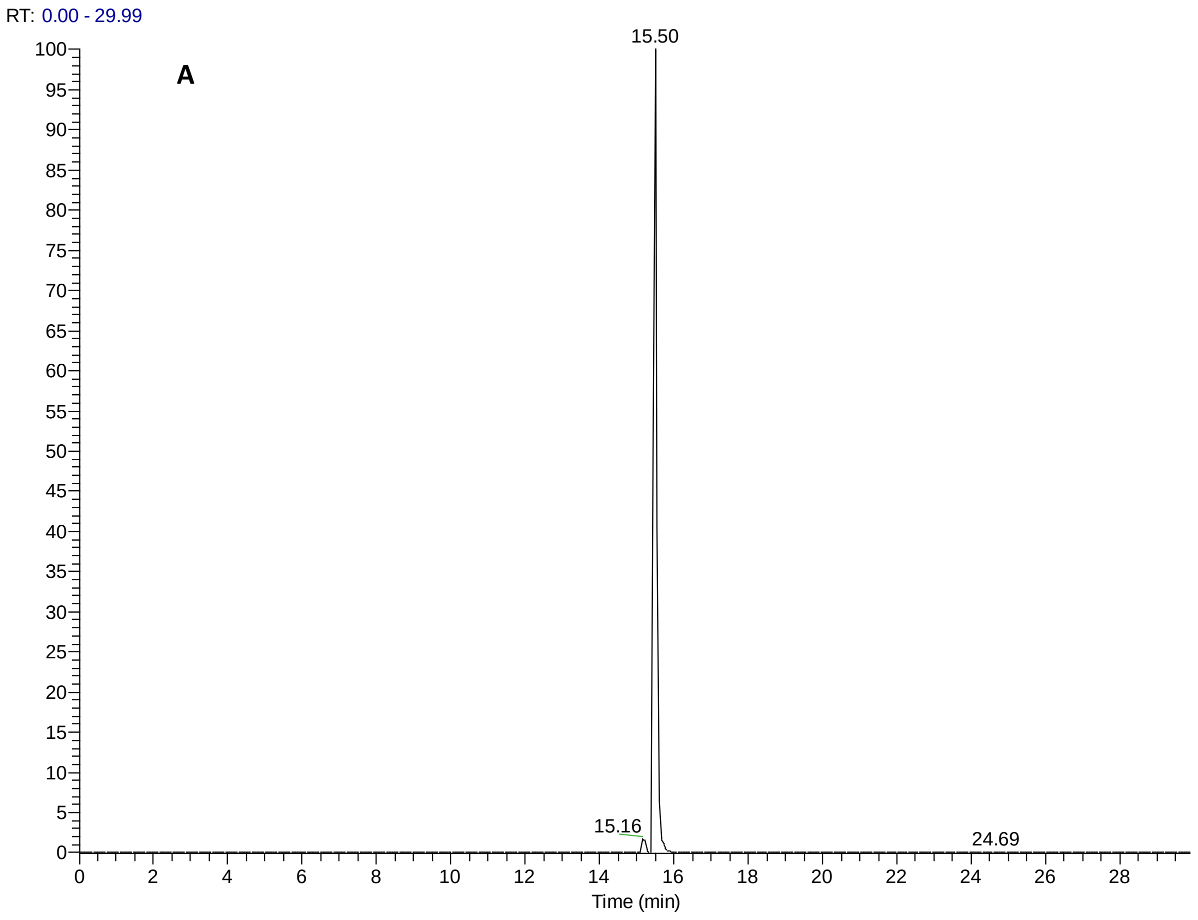

RT: 0.00 - 29.99
A
15.50
15.16
24.69
Time (min)

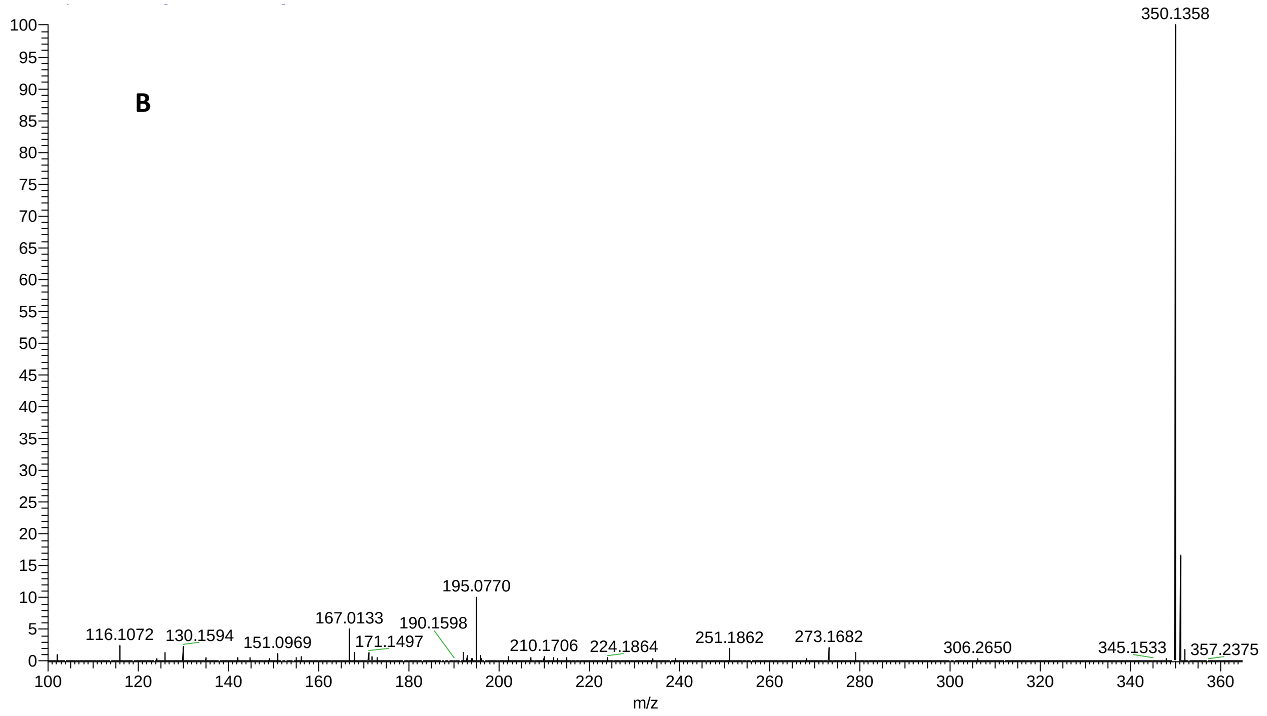

B
350.1358
195.0770
167.0133
190.1598
116.1072
130.1594
151.0969
171.1497
210.1706
224.1864
251.1862
273.1682
306.2650
345.1533
357.2375
m/z

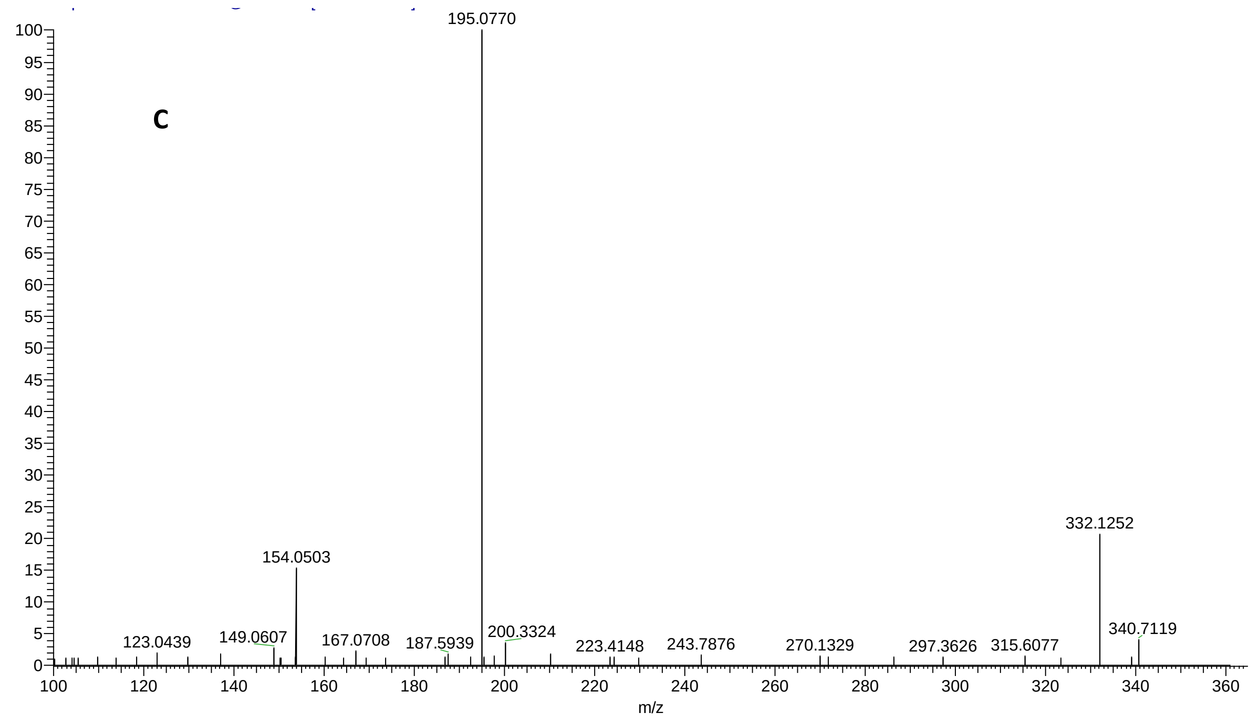

Figure S11. LC-HRMS analyses of sample **2** on an LTQ Orbitrap XL™ Hybrid FT Mass Spectrometer system: (**A**) Extracted ion Chromatogram (XIC) obtained by selecting the ion at *m/z* 350.1344, (**B**) relevant HRMS spectrum and (**C**) relevant HRMS^2 spectrum (Collision Induced Dissociation mode) obtained by using the ion at *m/z* 350.1 as precursor.

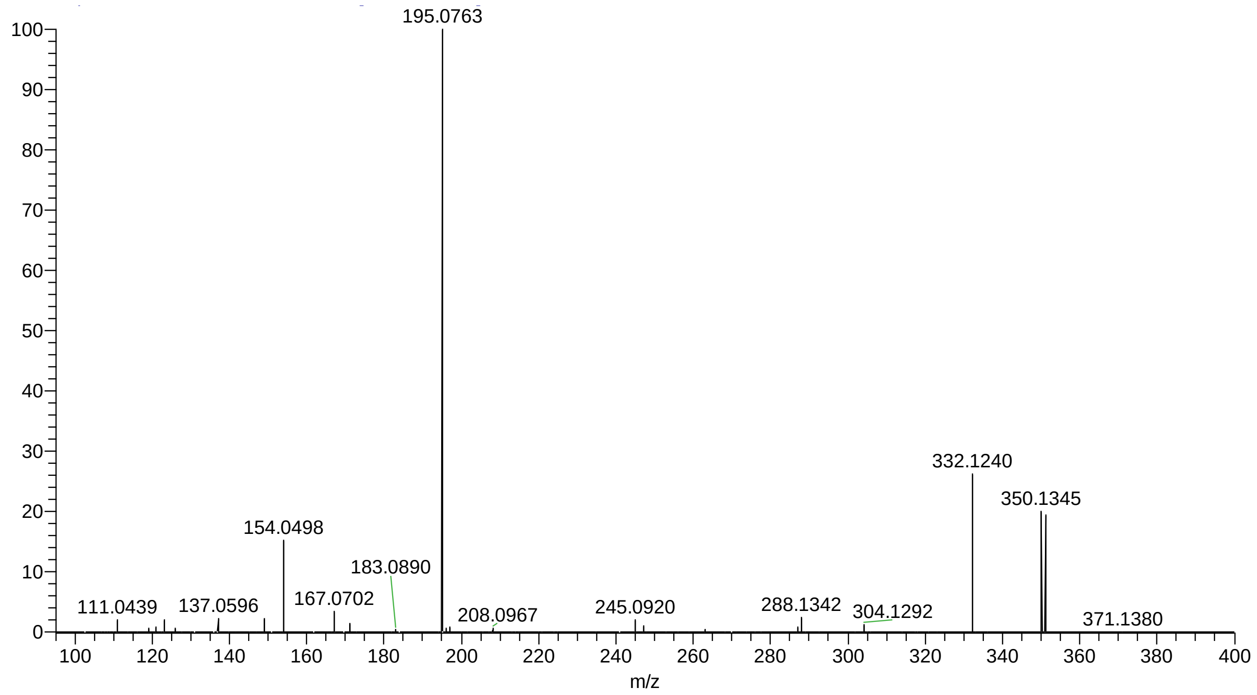

Figure S12. HR-MS² spectrum of *d***1RR** obtained by using the ion at *m/z* 350.1 as precursor. The sample was analyzed by direct infusion

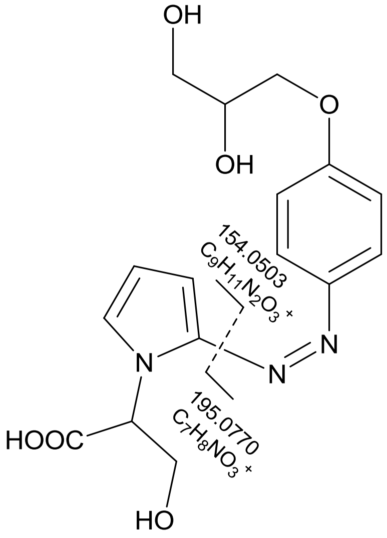

Figure S13. Fragmentation site occurring for *d***1RR** originating two key fragment ions.

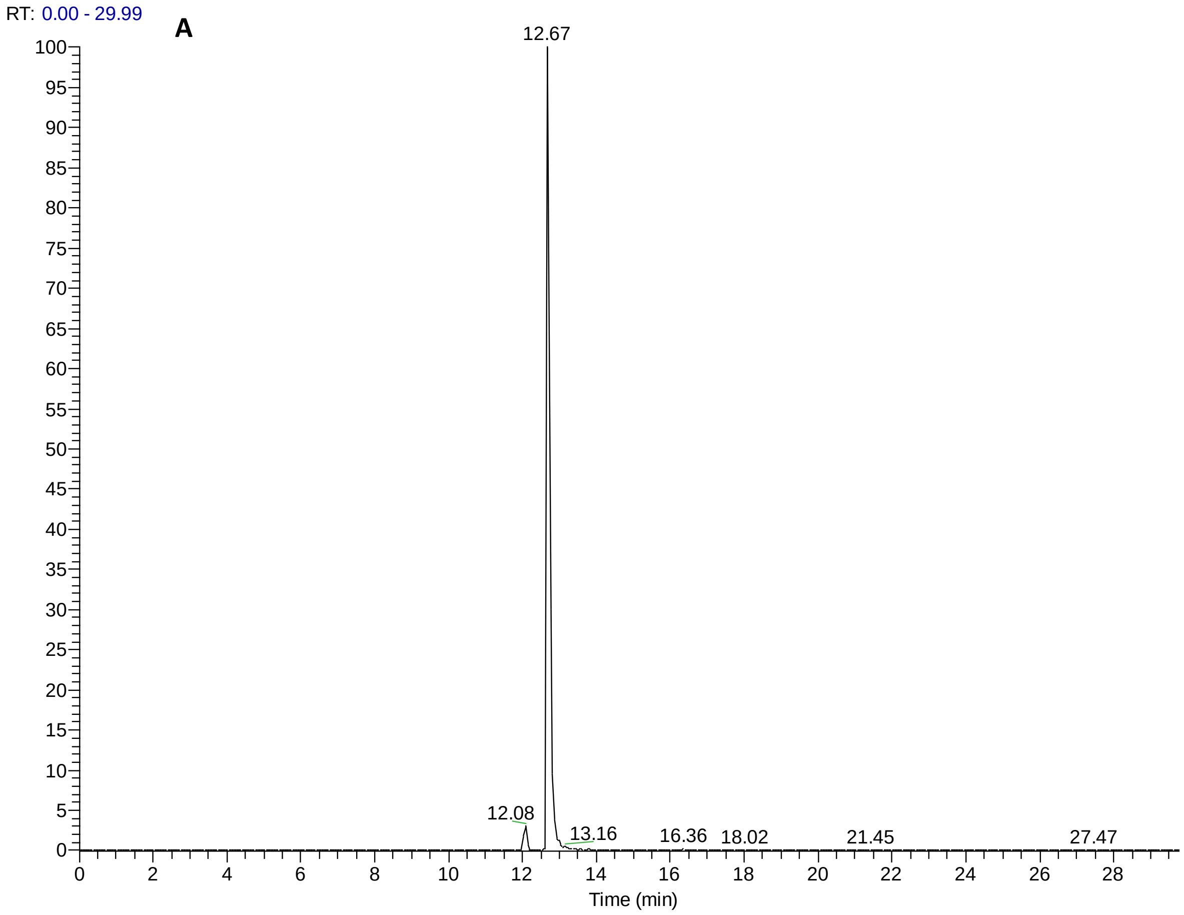
RT: 0.00 - 29.99
A
12.67
12.08
13.16
16.36
18.02
21.45
27.47
Time (min)

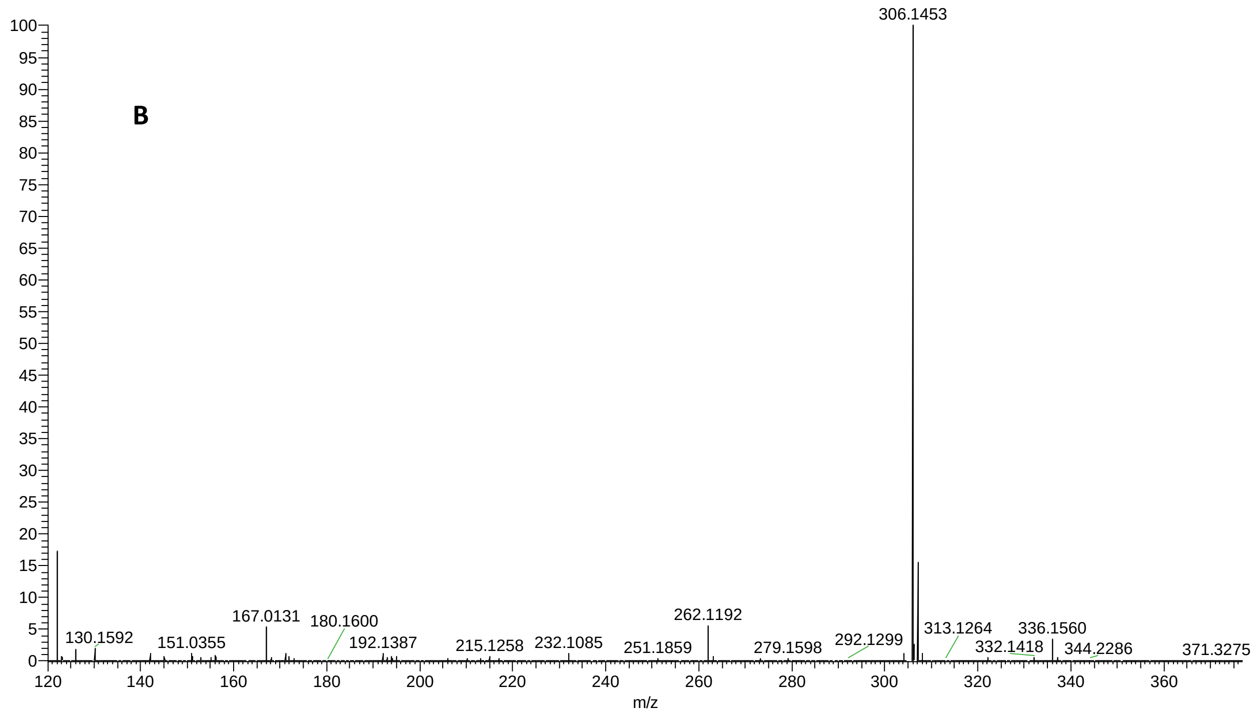
B
306.1453
130.1592
151.0355
167.0131
180.1600
192.1387
215.1258
232.1085
251.1859
262.1192
279.1598
292.1299
313.1264
332.1418
336.1560
344.2286
371.3275
m/z

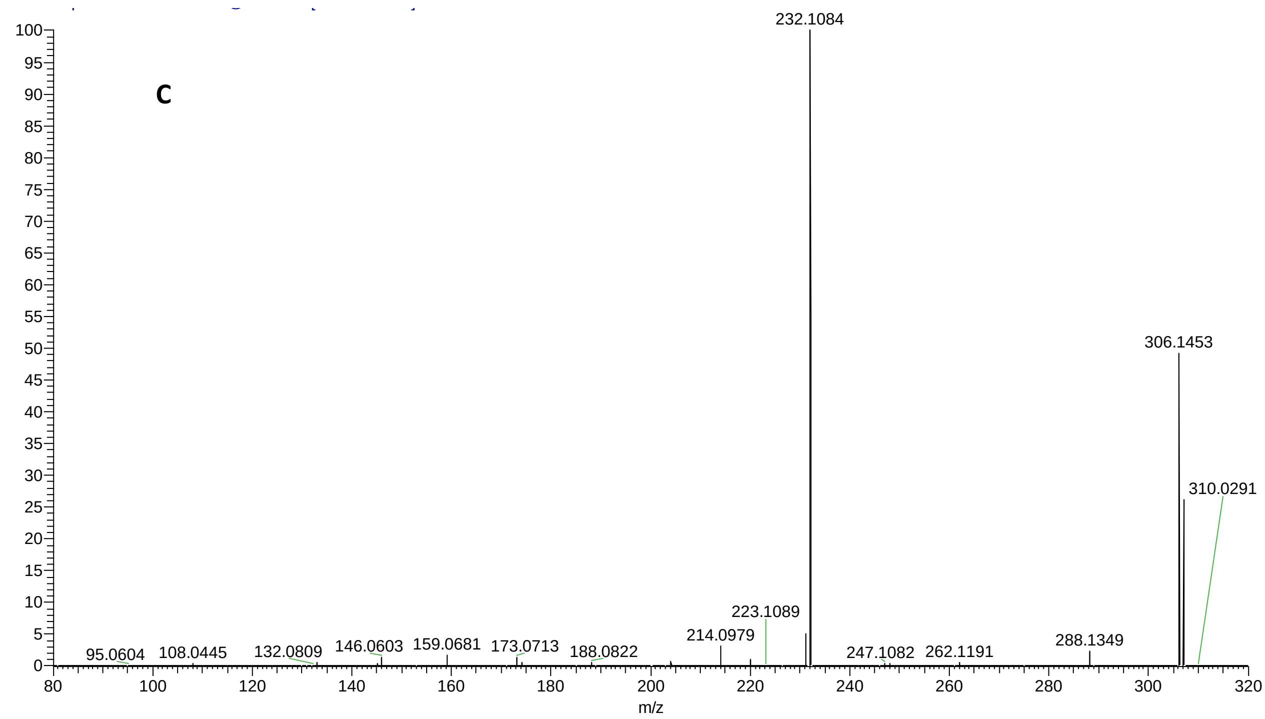

Figure S14. LC-HRMS analyses for sample **1** on an LTQ Orbitrap XL™ Hybrid FT Mass Spectrometer system: (**A**) Extracted ion Chromatogram (XIC) of the ion at *m*/*z* 306.1453, named compound **1**, (**B**) associated HRMS spectrum and (**C**) associated HRMS² spectrum (Collision Induced Dissociation mode) obtained by using the ion at *m*/*z* 306.1 as precursor.

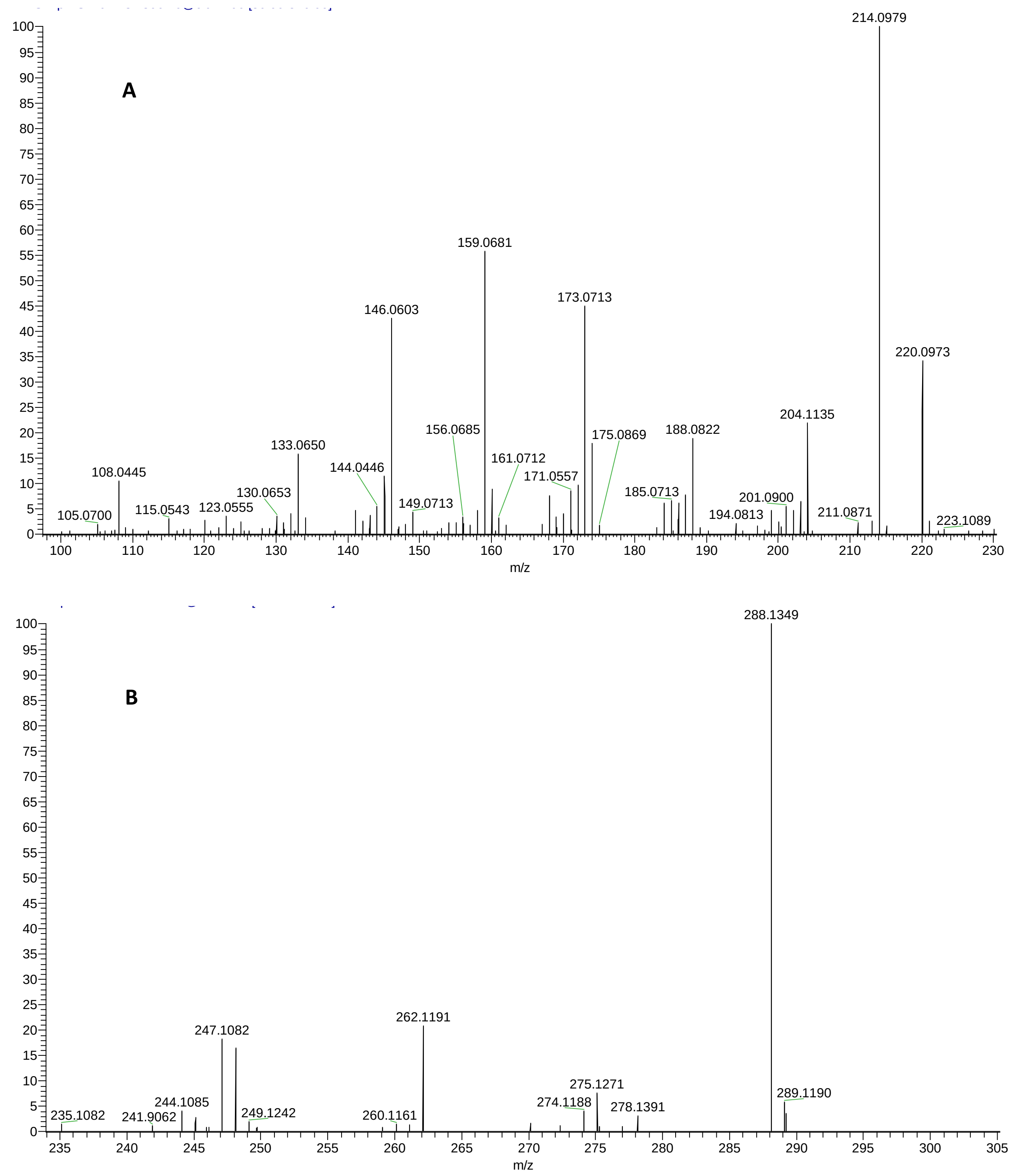

Figure S15. (**A**) Enlargement range *m/z* 90-230 and (**B**) *m/z* 230-305 of the LC-HRMS² spectrum of the compound **1**.

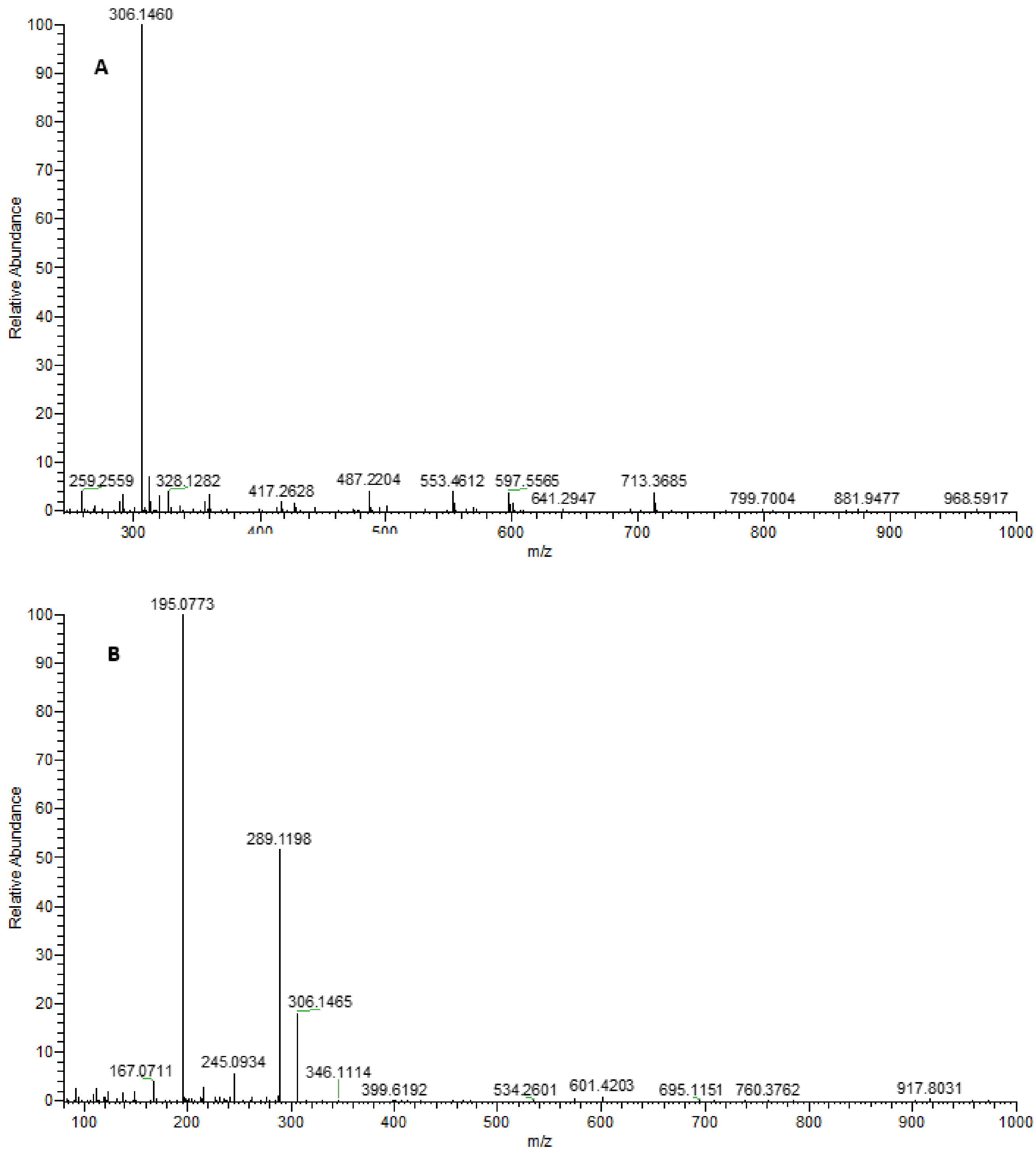

Figure S16. (**A**) HRMS spectrum of compound **2** and (**B**) relevant HRMS^2 spectrum obtained by using the ion at *m/z* 306.1 as precursor**.** The sample was analyzed by direct infusion.

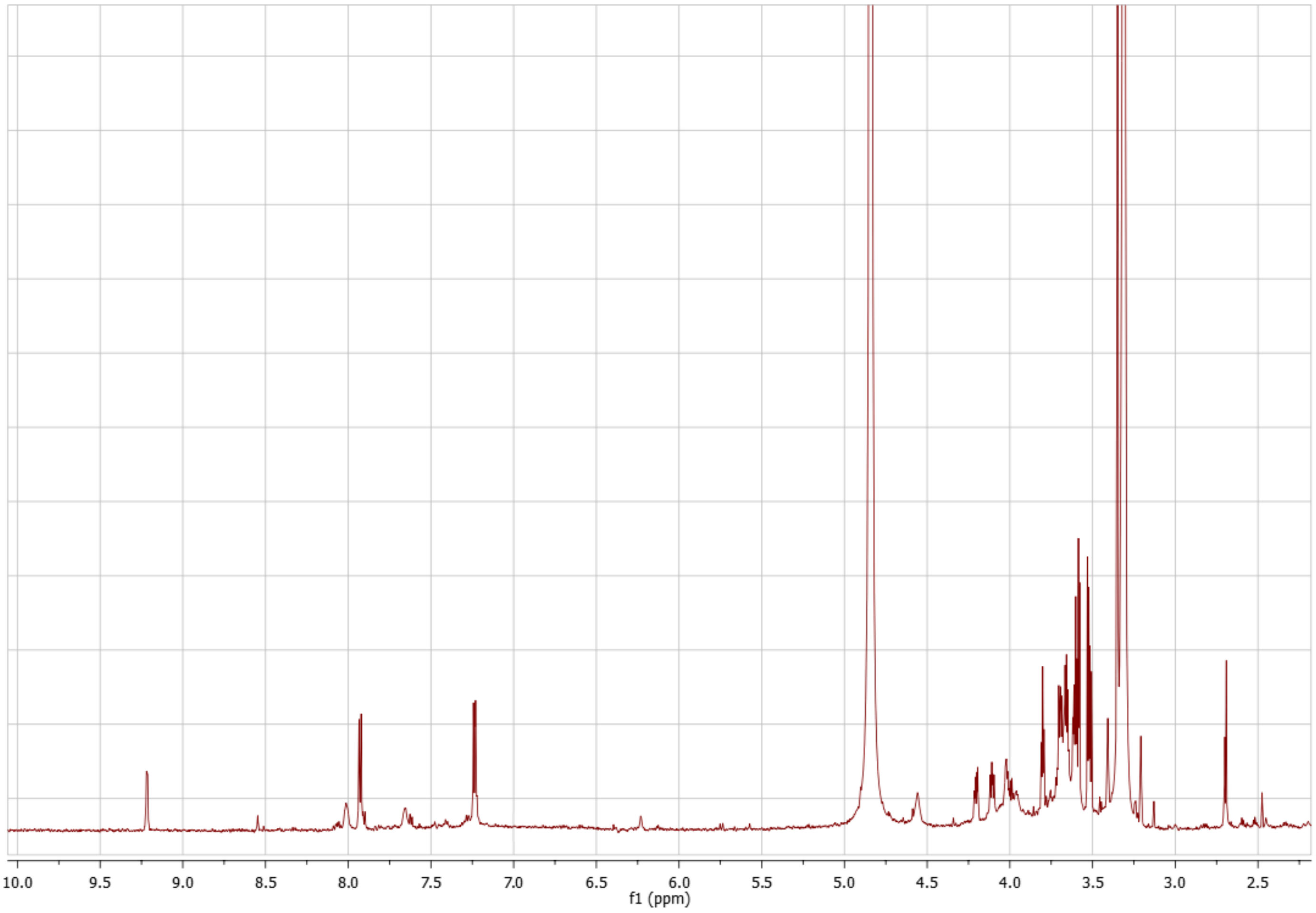

Figure S17. ^{1}H NMR spectrum of compound **1** in CD_3OD**.**

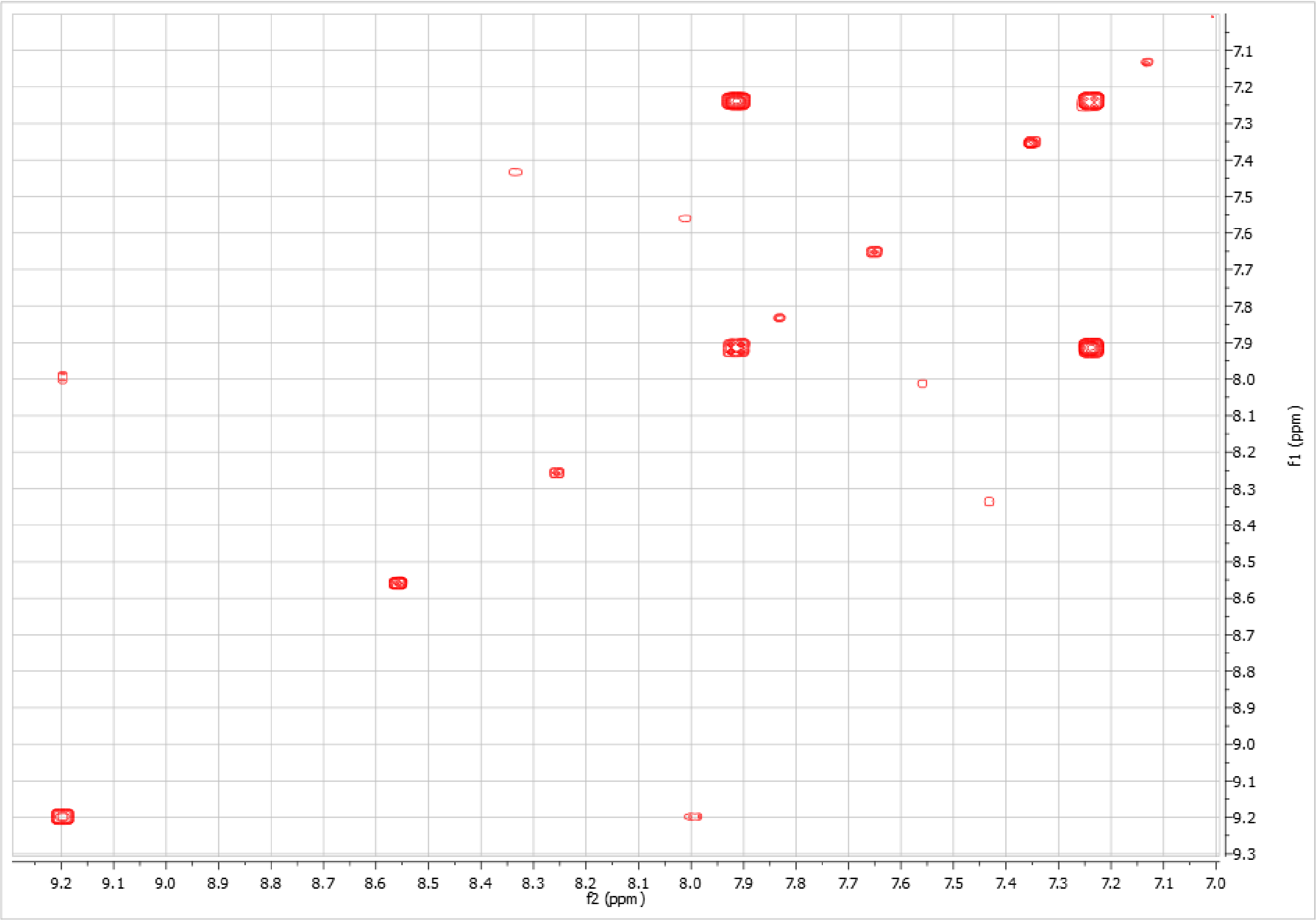

Figure S18. Low-field enlargement of ^{1}H-^{1}H COSY NMR spectrum of compound **1** in CD_3OD.

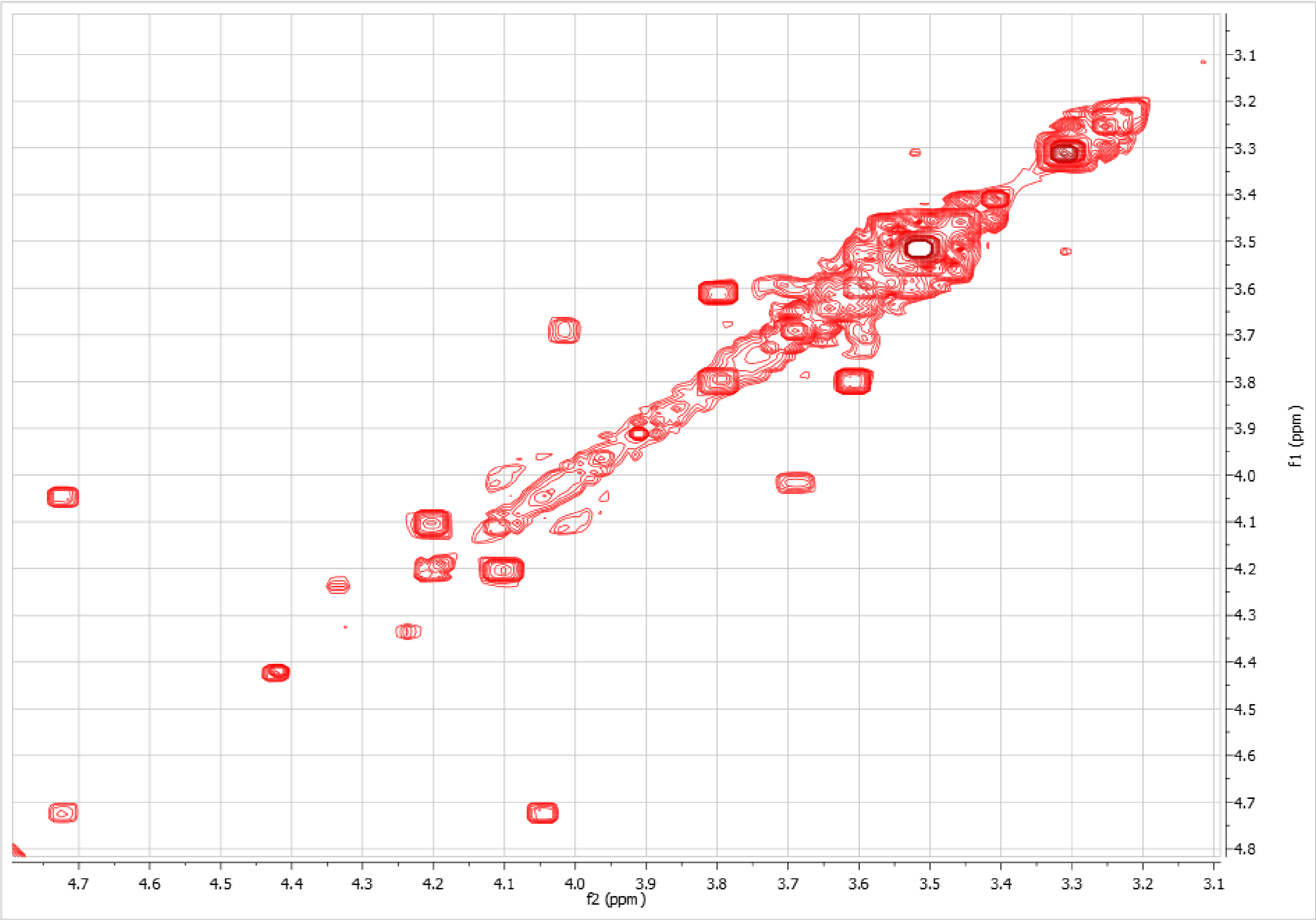

Figure S19. High-field enlargement of ^{1}H-^{1}H COSY NMR spectrum of compound **1** in CD_3OD**.**

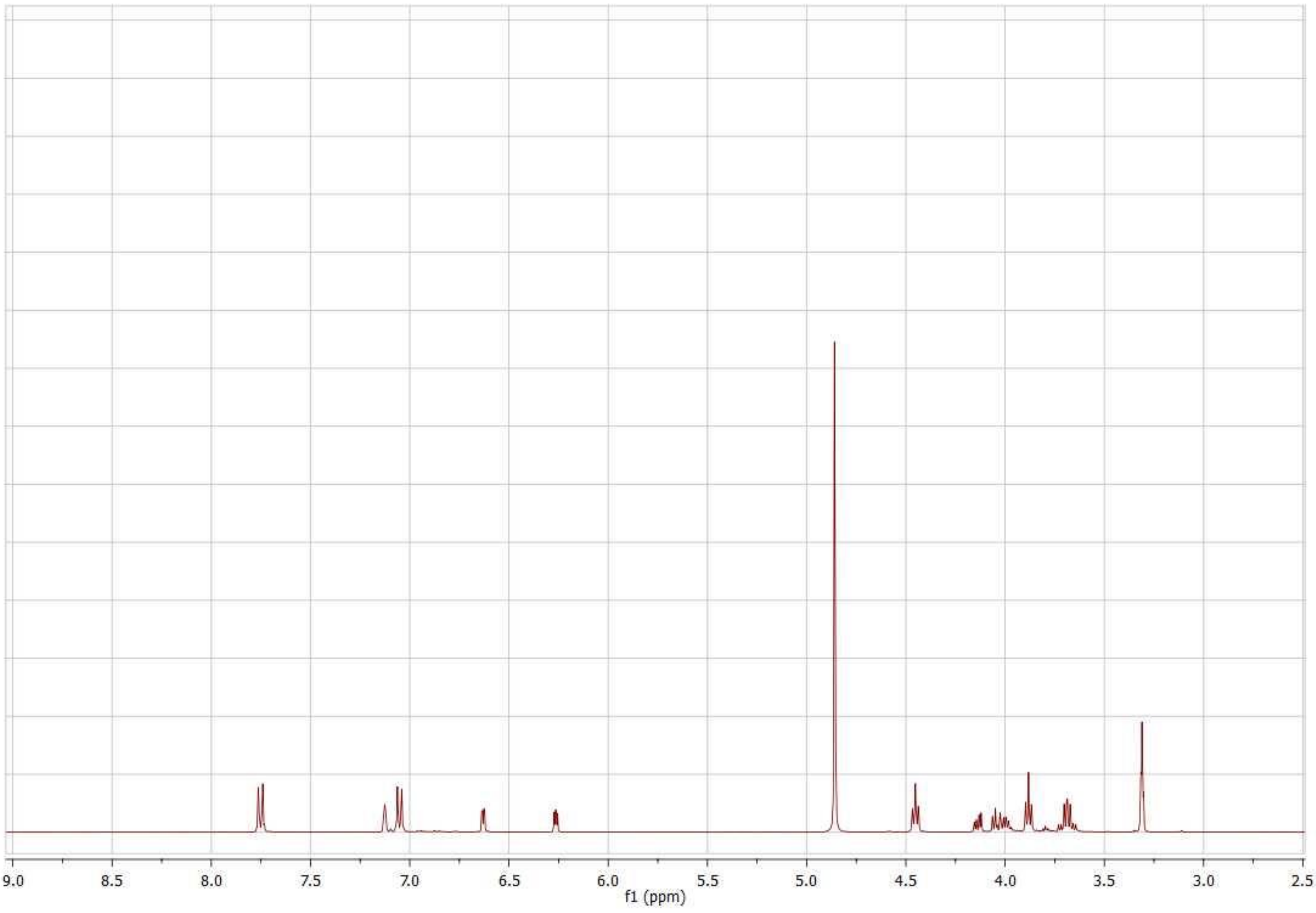

Figure S20. ^{1}H NMR spectrum of compound **2** in CD_3OD.

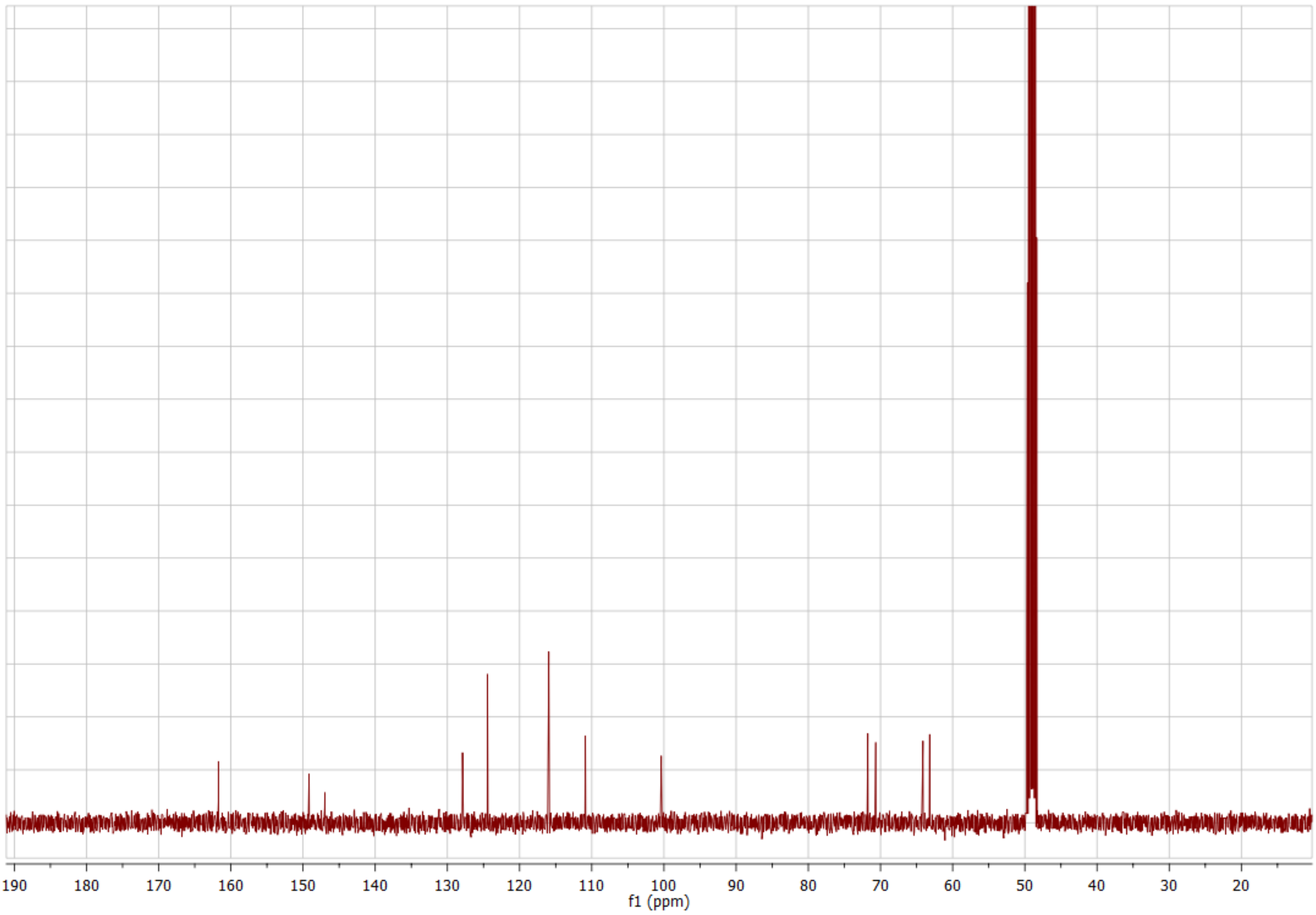

Figure S21. ^{13}C NMR spectrum of compound **2** in CD_3OD.

Table S1. All the apparent rate constants as a function of [H+] are fitted using the formula:

$$y = \frac{b + c\,x}{a + x}$$

			a*		b*		c*	
			Value $x10^{-7}$	SE** $x10^{-7}$	Value $x10^{-8}$	SE** $x\ 10^{-9}$	Value $x10^{-2}$	SE** $x\ 10^{-2}$
*d***1RR**	*trans* → *cis*	340 nm	15.5	5.99	9.19	41.8	35.9	5.67
		400 nm	24.0	2.46	14.97	14.2	1.88	3.07
	cis → *trans*	340 nm	30.0	1.10	2.45	10.2	27.8	3.41
		400 nm	8.18	2.45	0.115	5.25	23.8	2.52
*d***1RS**	*trans* → *cis*	340 nm	33.8	6.98	25.7	47.3	2.62	5.46
		400 nm	116	72.0	57.8	330	156	121
	cis → *trans*	340 nm	30.6	5.01	0.440	6.33	52.9	5.47
		400 nm	78.9	35.5	9.19	42.78	97.6	59.8

*All data are reported as absolute values. ** Standard Error on each value.

According to references in the manuscript [27],

$b=k_{T\rightarrow C}$ x K_aT ;

$c=k_{TH+\rightarrow CH+}$

and $a=K_aT$

where $k_{T\rightarrow C}$ is the rate constant for the *trans*→*cis* isomerization; K_aT is the equilibrium constant for *trans*H^+→ *trans*+ H^+; c is the rate constant for the reaction *trans*H^+→*cis*H^+ under irradiation, whereas all these parameters are connected to the *cis* form during the thermal decay.

Table S2. Calculated percentages of ionic species of *d***1RR** and *d***1RS** in the pH range 1.0-8.0.

**pH**	**% Ionic forms**			
	**Protonated**	**Zwitterionic**	**Anionic**	**Neutral**
**8.0**	0	5	95	0
**7.5**	0	14	86	0
**7.0**	0	33	67	0
**6.7**	0	50	50	0
**6.5**	0	61	39	0
**6.0**	0	83	17	0
**5.7**	0	91	9	0
**5.0**	0	98	2	0
**4.0**	0	100	0	0
**3.0**	4	96	0	0
**2.0**	28	72	0	0
**1.0**	80	20	0	0

Table S3. ΔE_{GM} values (kcal/mol) and torsion angle values (degrees) of the MM conformers of trans isomer of *d***1RR.**

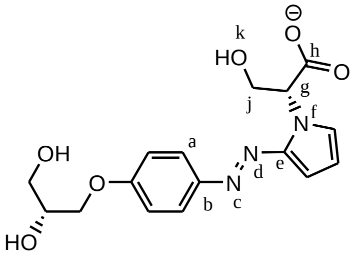

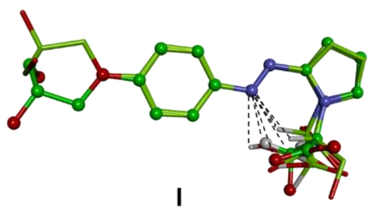

I

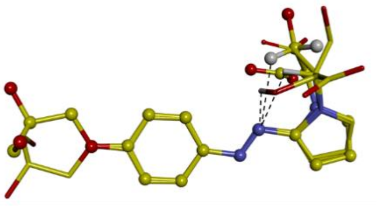

II

Sub-family	ΔE_{GM} (kcal/mol)[a]	(%)	Torsion Angles (°)[b]					Intramolecular interactions
			$\tau 1^c$	$\tau_{N=N}{}^d$	$\tau 2^e$	$\tau 3^f$	$\tau 4^g$	
**Ia**	0.00-4.96	21.5	-4.94	179.90	-4.30	-63.11	169.89	CH_2 (j) – N (c)
**Ib**	0.92-4.96	16.3	0.61	-179.89	-9.04	95.83	176.13	CH (g) – N (c)
**IIc**	1.37-4.98	18.2	2.67	-179.45	155.49	-62.51	176.33	CH_2 (g) – N (d)
**IId**	2.40-4.94	20.2	-1.85	179.66	-156.07	100.29	177.41	CH (g) – N (d)
**Ie**	2.31-4.99	10.7	-6.35	179.95	-0.20	-59.94	-74.41	OH (k) – N(c)
**IIf**	3.62-4.96	5.8	-2.06	179.49	-155.47	-60.25	-68.39	OH (k) – N(d)
**Ig**	3.80-4.93	3.8	-0.43	-179.94	14.39	132.41	-64.48	CH (g) – N (c)<br>CH_2 (j) – N (c)
**Ih**	3.91-4.96	3.2	0.04	180.00	15.19	130.08	62.22	OH (k) – N(c)<br>CH (g) – N (c)

[a]The values reported refer to the lowest and the highest energy conformers of the sub-family. [b]The values reported refer to the lowest energy conformer of the sub-family. [c]τ_1 torsion angle is defined by a, b, c, and d atoms. [d]$\tau_{N=N}$ torsion angle is defined by b, c, d, and e atoms. [e]$\tau 2$ torsion angle is defined by c, d, e, and f atoms. [f]$\tau 3$ torsion angle is defined by e, f, g, and h atoms. [g]$\tau 4$ torsion angle is defined by f, g, j, and k atoms.

Table S4. ΔE_{GM} values (kcal/mol) and torsion angle values (degrees) of the MM conformers of cis isomer *d***1RR**.

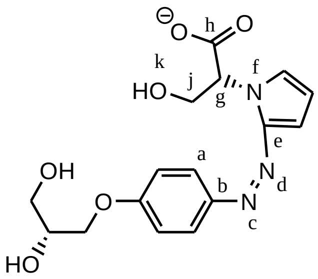

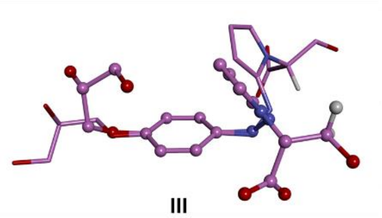

III

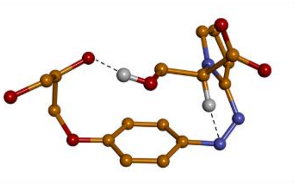

IV

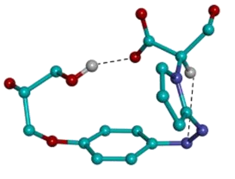

V

Sub-family	ΔE_{GM} (kcal/mol)[a]	Number of conf.(%)	Torsion Angles (°)[b]					Intramolecular interactions
			$\tau 1$[c]	$\tau_{N=N}$[d]	$\tau 2$[e]	$\tau 3$[f]	$\tau 4$[g]	
**IVj**	0.00-4.99	27.2	51.76	4.78	65.08	100.61	173.62	CH_2OH – OH<br>CH (g) – N (c)
**IIIk**	0.81-4.96	36.0	-60.80	-3.80	127.57	-63.57	174.46	CH_2 (j) – N (d)
**IIIi**	1.64-5.00	24.2	53.89	4.03	-128.88	105.70	176.51	CH (g) – N (d)
**V**L	1.14-4.96	12.6	-60.49	-2.97	-86.03	124.89	177.97	COO- OH<br>CH (g) – N (c)

[a]The values reported refer to the lowest and the highest energy conformers of the sub-family. [b]The values reported refer to the lowest energy conformer of the sub-family. [c]τ_1 torsion angle is defined by a, b, c, and d atoms. [d]$\tau_{N=N}$ torsion angle is defined by b, c, d, and e atoms. [e]$\tau 2$ torsion angle is defined by c, d, e, and f atoms. [f]$\tau 3$ torsion angle is defined by e, f, g, and h atoms. [g]$\tau 4$ torsion angle is defined by f, g, j, and k atoms.

Table S5. ΔE_{GM} values (kcal/mol) and torsion angle values (degrees) of the MM conformers of trans isomer of *d***1RS.**

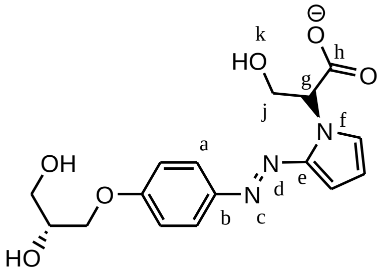

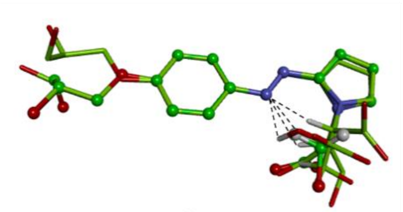

I

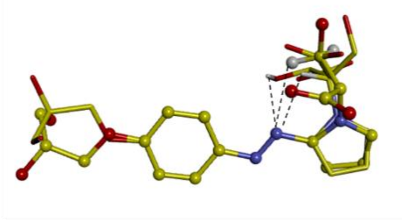

II

Sub-family	ΔE_{GM} (kcal/mol)[a]	Number of conf. (%)	Torsion Angles (°)[b]					Intramolecular interactions
			$\tau 1^{c}$	$\tau_{N=N}{}^{d}$	$\tau 2^{e}$	$\tau 3^{f}$	$\tau 4^{g}$	
**Ia**	0.00-4.98	31.9	5.68	-179.89	4.51	63.03	-169.90	CH_2 (j) – N (c)
**Ib**	0.99-4.97	12.8	0.51	179.88	9.00	-95.82	-176.13	CH (g) – N (c)
**IIc**	1.38-4.99	20.9	-2.64	179.46	-155.56	62.53	-176.33	CH_2 (g) – N (d)
**IId**	2.40-4.97	13.0	2.07	-179.66	156.58	-100.39	-177.42	CH (g) – N (d)
**Ie**	2.30-4.96	10.9	6.54	-179.93	0.36	59.89	74.38	OH (k) – N(c)
**IIf**	3.63-4.95	7.1	2.01	-179.49	155.41	60.28	68.40	OH (k) – N(d)
**Ig**	3.81-4.97	3.4	0.35	179.93	-14.40	-132.38	64.48	CH (g) – N (c)<br>CH_2 (j) – N (c)

[a]The values reported refer to the lowest and the highest energy conformers of the sub-family. [b]The values reported refer to the lowest energy conformer of the sub-family. [c]τ_1 torsion angle is defined by a, b, c, and d atoms. [d]$\tau_{N=N}$ torsion angle is defined by b, c, d, and e atoms. [e]τ2 torsion angle is defined by c, d, e, and f atoms. [f]τ3 torsion angle is defined by e, f, g, and h atoms. [g]τ4 torsion angle is defined by f, g, j, and k atoms.

Table S6. ΔE_{GM} values (kcal/mol) and torsion angle values (degrees) of the MM conformers of cis isomer *d***1RS**.

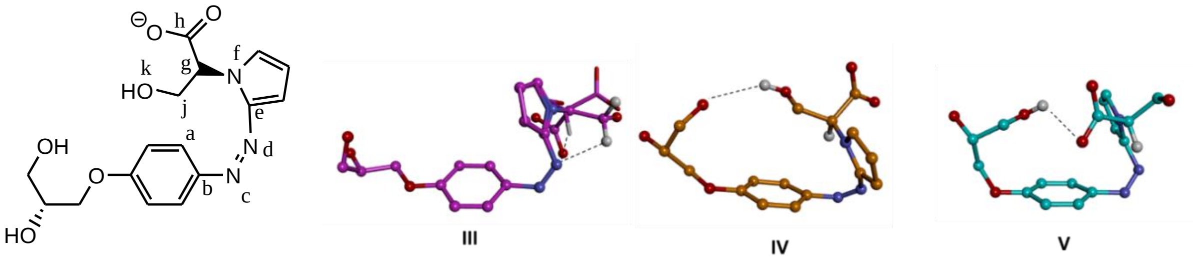

III IV V

Sub-family	ΔE_{GM} (kcal/mol)[a]	Number of conf.(%)	Torsion Angles (°)[b]					Intramolecular interactions
			$\tau1^{c}$	$\tau_{N=N}{}^{d}$	$\tau2^{e}$	$\tau3^{f}$	$\tau4^{g}$	
**IVj**	0.00-4.99	4.4	49.85	4.96	-63.61	100.55	175.134	CH_2OH – OH<br>CH (g) – N (c)
**IIIk**	0.13-4.97	50.3	55.29	4.06	127.70	64.12	-176.13	CH_2 (j) – N (d)
**V$_L$**	0.40-5.00	29.1	59.90	3.05	85.12	125.21	-179.40	COO- OH<br>CH (g) – N (c)
**IIIi**	1.17-4.98	16.2	57.71	4.16	120.84	104.10	-177.56	CH (g) – N (d)

[a]The values reported refer to the lowest and the highest energy conformers of the sub-family. [b]The values reported refer to the lowest energy conformer of the sub-family. [c]τ_1 torsion angle is defined by a, b, c, and d atoms. [d]$\tau_{N=N}$ torsion angle is defined by b, c, d, and e atoms. [e]$\tau2$ torsion angle is defined by c, d, e, and f atoms. [f]$\tau3$ torsion angle is defined by e, f, g, and h atoms. [g]$\tau4$ torsion angle is defined by f, g, j, and k atoms.

Table S7. ΔE_{GM} values (kcal/mol) and torsion angle values (degrees) of the MM conformers of *d***1RR** in the zwitterionic form.

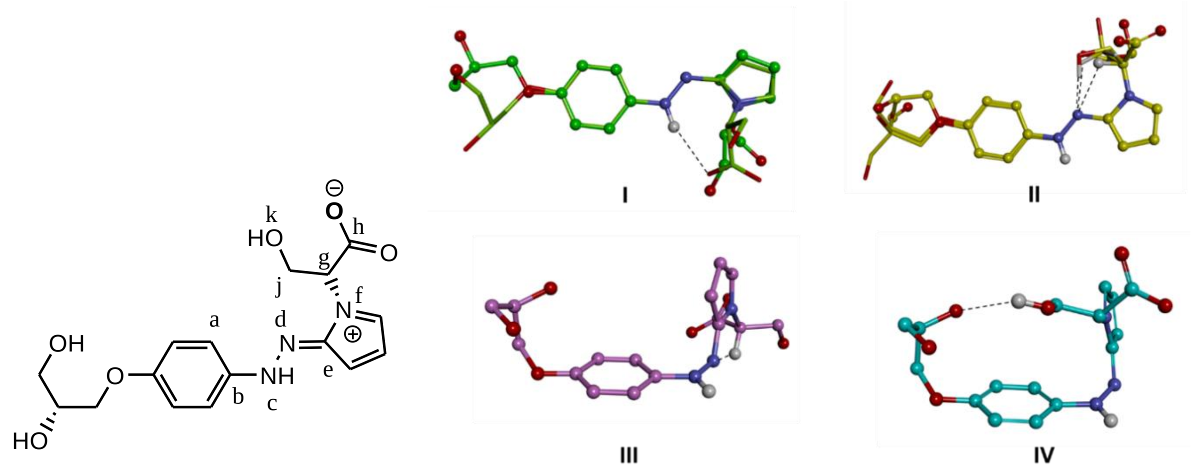

**Sub-family**	**ΔE_{GM} (kcal/mol)**[a]	**Number of conf.(%)**	**Torsion Angles (°)**[b]					**Intramolecular interactions**
			$\tau 1^{c}$	$\tau_{N=N}{}^{d}$	$\tau 2^{e}$	$\tau 3^{f}$	$\tau 4^{g}$	
**IId**	0.00-4.98	66.0	0.00	179.74	179.99	122.25	176.88	CH (g) – N (d)
**IIc**	1.89-4.89	16.1	-0.26	-179.94	178.66	-63.89	176.76	CH_2 (g) – N (d)
**I**	2.70-4.99	14.1	-0.44	-176.46	28.47	134.09	175.98	-
**IIIi**	3.96-4.95	1.1	1.62	76.65	-170.50	115.84	176.91	CH (g) – N (d)
**Im**	4.01-4.79	0.4	0.60	175.55	-37.58	109.98	176.71	COO- NH
**IVn**	4.62-4.98	1.5	4.67	61.95	29.93	121.07	175.21	CH_2OH – OH
**IIf**	4.63-4.87	0.8	-0.09	179.04	179.63	-58.55	-69.31	OH (k) – N(d)

[a]The values reported refer to the lowest and the highest energy conformers of the sub-family. [b]The values reported refer to the lowest energy conformer of the sub-family. [c]τ_1 torsion angle is defined by a, b, c, and d atoms. [d]$\tau_{N=N}$ torsion angle is defined by b, c, d, and e atoms. [e]τ2 torsion angle is defined by c, d, e, and f atoms. [f]τ3 torsion angle is defined by e, f, g, and h atoms. [g]τ4 torsion angle is defined by f, g, j, and k atoms.

Table S8. ΔE_{GM} values (kcal/mol) and torsion angle values (degrees) of the MM conformers of *d***1RS** in the zwitterionic form.

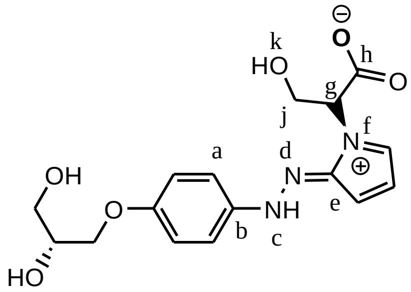

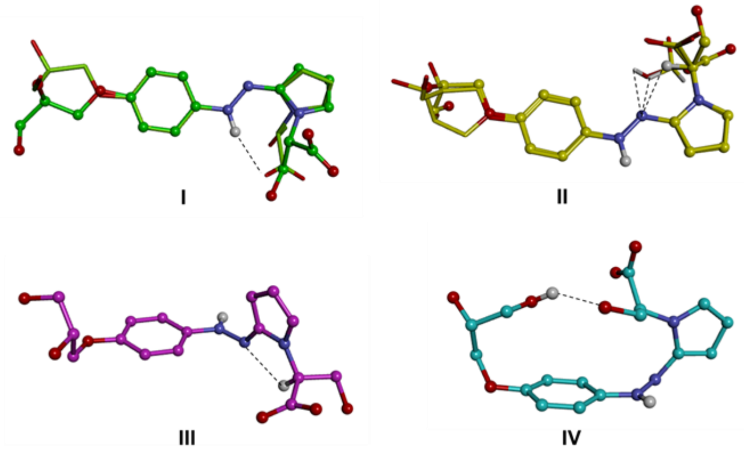

Sub-family	ΔE_{GM} (kcal/mol)[a]	Number of conf.(%)	Torsion Angles (°)[b]					Intramolecular interactions
			$\tau1^{c}$	$\tau_{N=N}{}^{d}$	$\tau2^{e}$	$\tau3^{f}$	$\tau4^{g}$	
**IId**	0.00-4.88	35.83	0.00	-179.73	-179.97	-122.58	-176.80	CH (g) – N (d)
**IIc**	1.75-4.78	36.70	0.26	179.74	178.75	63.97	176.63	CH_2 (g) – N (d)
**I**	2.64-4.96	15.38	0.50	176.60	-27.87	134.37	-175.81	-
**IIIi**	3.50-4.88	0.56	-0.90	-80.05	170.95	-115.99	-176.787	CH (g) – N (d)
**IIf**	4.54-4.97	2.72	0.17	-178.79	-179.48	58.01	66.53	OH (k) – N(d)
**Im**	3.62-4.77	8.54	-0.50	-175.56	38.00	-111.00	-174.51	COO- NH
**IVn**	4.04-4.73	0.28	6.91	120.77	-50.56	-130.13	-172.43	CH_2OH – OH

[a]The values reported refer to the lowest and the highest energy conformers of the sub-family. [b]The values reported refer to the lowest energy conformer of the sub-family. [c]τ_1 torsion angle is defined by a, b, c, and d atoms. [d]$\tau_{N=N}$ torsion angle is defined by b, c, d, and e atoms. [e]$\tau2$ torsion angle is defined by c, d, e, and f atoms. [f]$\tau3$ torsion angle is defined by e, f, g, and h atoms. [g]$\tau4$ torsion angle is defined by f, g, j, and k atoms.

Table S9. ΔG_{GM} values (kcal/mol) and torsion angle values (degrees) of the DFT trans isomers of *d***1RS** and *d***1RR** in the anionic form.

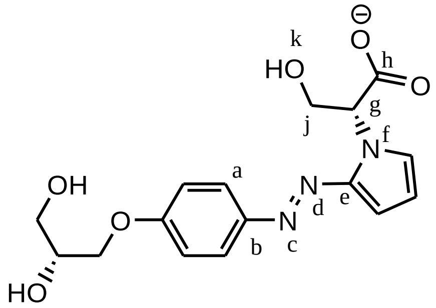

Cmp	ΔG_{GM} (kcal/mol)	Torsion Angles (°)					Intramolecular interaction
		τ1[a]	$\tau_{N=N}$[b]	τ2[c]	τ3[d]	τ4[e]	
*d***1RS**	0.00	1.88	179.99	0.29	61.71	-172.13	CH_2 (j) – N (c)
	1.32	-0.97	-179.99	0.28	63.52	-62.41	CH_2 (j)-N (c)
*d***1RR**	0.00	-2.30	179.28	-1.51	-62.72	63.22	CH_2 (j) -N (c)
	4.93	4.50	-179.37	4.71	122.89	173.84	CH (g) -N (c)

[a]τ_1 torsion angle is defined by a, b, c, and d atoms. [b]$\tau_{N=N}$ torsion angle is defined by b, c, d, and e atoms. [c]τ2 torsion angle is defined by c, d, e, and f atoms. [d]τ3 torsion angle is defined by e, f, g, and h atoms. [e]τ4 torsion angle is defined by f, g, j, and k atoms.

Table S10. ΔG_{GM} values (kcal/mol) and torsion angle values (degrees) of the DFT cis isomer of *d***1RS** and *d***1RR** in the anionic form.

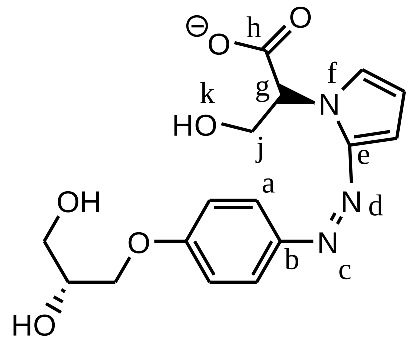

Cmp	ΔG_{GM} (kcal/mol)	Torsion Angles (°)					Intramolecular interaction
		$\tau 1^{a}$	$\tau_{N=N}{}^{b}$	$\tau 2^{c}$	$\tau 3^{d}$	$\tau 4^{e}$	
***d*1RS**	0.00	80.58	4.10	-177.42	59.78	-171.24	CH-π
	5.31	42.99	11.82	43.83	-148.54	-168.65	COO- OH
	7.55	-34.60	-12.60	-45.16	-91.84	-171.90	CH (g) – N (c)
***d*1RR**	0.00	92.60	0.60	-179.64	117.40	172.12	CH-π
	8.24	32.92	12.66	46.95	91.67	171.42	OH – Ph
	8.99	-44.36	-12.12	-47.45	145.61	169.09	COO- OH

[a]τ_1 torsion angle is defined by a, b, c, and d atoms. [b]$\tau_{N=N}$ torsion angle is defined by b, c, d, and e atoms. [c]$\tau 2$ torsion angle is defined by c, d, e, and f atoms. [d]$\tau 3$ torsion angle is defined by e, f, g, and h atoms. [e]$\tau 4$ torsion angle is defined by f, g, j, and k atoms.

Table S11. ΔG$_{GM}$ values (kcal/mol) and torsion angle values (degrees) of the DFT conformers of *d***1RS** and *d***1RR** in the zwitterionic form.

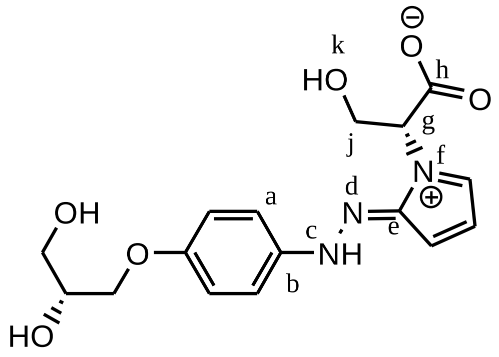

Cmp	ΔG$_{GM}$ (kcal/mol)	Torsion Angles (°)					Intramolecular interaction
		τ1[a]	τ$_{N=N}$[b]	τ2[c]	τ3[d]	τ4[e]	
***d*1RS**	0.00	9.94	-176.77	15.33	-94.72	-174.35	COO- NH
	1.22	0.17	-179.24	-179.70	-122.09	-171.38	CH (g) – N (d)
	19.92	34.28	23.43	18.84	-160.02	-171.50	COO$^-$ OH
***d*1RR**	0.00	7.97	-177.66	9.34	-78.55	173.78	COO- NH
	0.24	0.42	-179.90	-179.79	126.33	-68.48	CH (g) – N (d)
	17.56	-43.23	-16.84	-12.91	143.74	168.90	COO$^-$ OH
	18.60	30.29	22.88	17.24	85.56	170.30	CH_2OH-OH

[a]τ_1 torsion angle is defined by a, b, c, and d atoms. [b]$\tau_{N=N}$ torsion angle is defined by b, c, d, and e atoms. [c]τ2 torsion angle is defined by c, d, e, and f atoms. [d]τ3 torsion angle is defined by e, f, g, and h atoms. [e]τ4 torsion angle is defined by f, g, j, and k atoms.

Table S12. Bond length values (Å) of the DFT conformers of *d***1RR** and *d***1RS.**

**Cmp**	**Form**	**Isomer**	**Bond length (Å)**[a]		
			C (b) – N (c)	N (c) – N (d)	N (d) – C (e)
***d*1RR**	Anionic	*trans*	1.414	1.272	1.370
	Anionic	*cis*	1.439	1.267	1.376
	Zwitterionic	-	1.408	1.280	1.341
***d*1RS**	Anionic	*trans*	1.414	1.271	1.372
	Anionic	*cis*	1.438	1.267	1.378
	Zwitterionic	-	1.405	1.284	1.341

[a] Average bond lengths: C-N (1.43 Å), C=N (1.38 Å), N-N (1.47 Å) and N=N (1.24 Å)

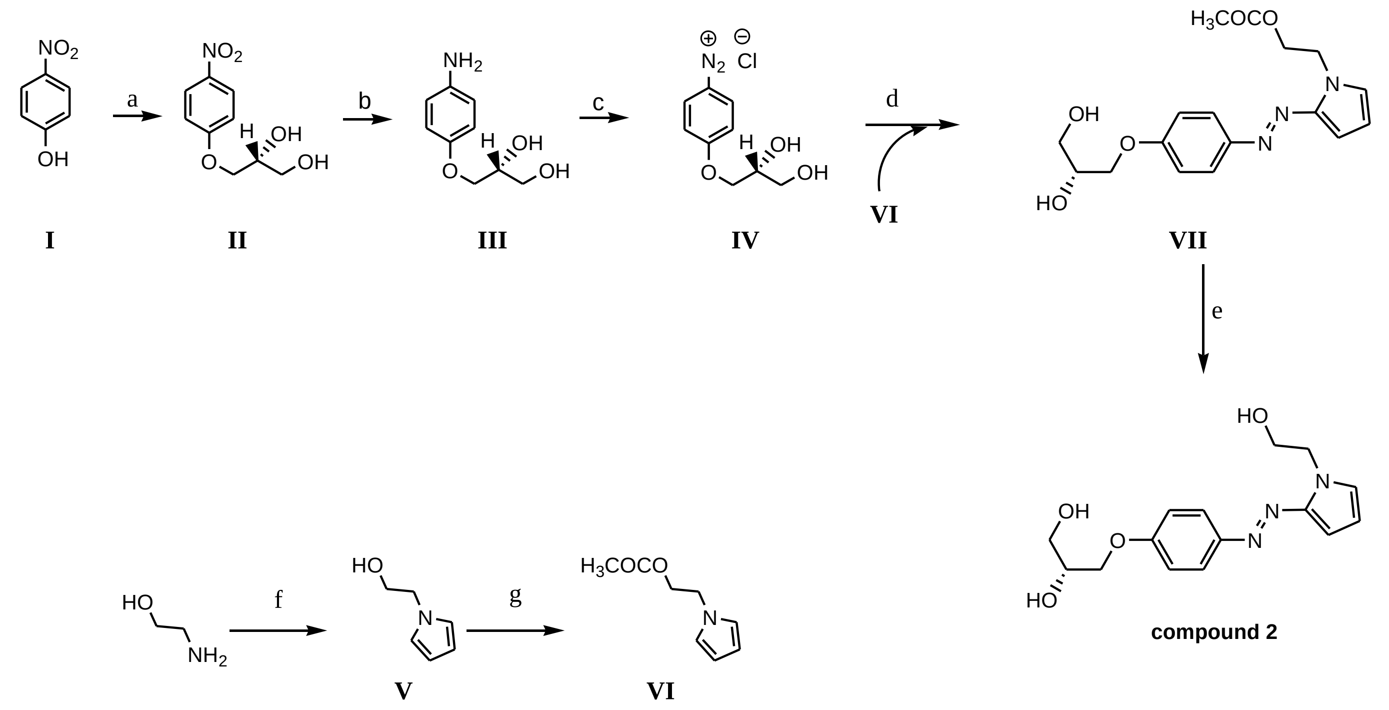

Scheme S1: Synthesis of compound **2**. Reagents and conditions: a) catalytic KOH, dry toluene, 30 min under reflux, then addition of *R* glycidol at r.t., and eating at 90°C for 24h. b) Pd/C, H_2 (P= 400 psi) in EtOH, 2 h, r.t. c) fluoroboric acid (water solution, 48%), EtOH,-15°C, then isoamyl nitrite, r.t., 12 h. d) acetic acid, sodium acetate 1 h, r.t. For more details see the reference [20] in the manuscript; e) KOH (1.2 mmol) in EtOH, 2h, rt. f) 2,5-dimethoxytetrahydrofuran, H_2O, reflux, 2h, then ethanolamine in DCM, 24h, rt, overnight; g) acetic anhydride, DCM, rt.
